# Different profiles of soil phosphorous compounds depending on tree species and availability of soil phosphorus in a tropical rainforest in French Guiana

**DOI:** 10.1186/s12870-024-04907-x

**Published:** 2024-04-12

**Authors:** Albert Gargallo-Garriga, Jordi Sardans, Joan Llusià, Guille Peguero, Marta Ayala-Roque, Elodie A. Courtois, Clément Stahl, Otmar Urban, Karel Klem, Pau Nolis, Miriam Pérez-Trujillo, Teodor Parella, Andreas Richter, Ivan A. Janssens, Josep Peñuelas

**Affiliations:** 1 Global Change Research Institute, The Czech Academy of Sciences, Belidla 986/4a, Brno, CZ-60300 Czech Republic; 2 Universitat Autònoma de Barcelona, Bellaterra, 08193 Spain; 3 Global Ecology Unit, CSIC, CREAF-CSIC-UAB, Bellaterra, 08193 Catalonia Spain; 4 CREAF, Cerdanyola del vallès, Barcelona, Catalonia 08193 Spain; 5 Centre of Excellence PLECO (Plants and Ecosystems), Department of Biology, University of Antwerp, Wilrijk, Belgium; 6 Laboratoire écologie, évolution, Interactions des Systèmes Amazoniens (LEEISA), Université de Guyane, CNRS, IFREMER, Cayenne, France; 7 UMR ECOFOG - Ecologie des forêts de Guyane, Kourou cedex, 97379 France; 8 Servei de Ressonància Magnètica Nuclear, Universitat Autònoma de Barcelona, Bellaterra, 08193 Spain; 9 Centre for Microbiology and Environmental Systems Science, University of Vienna, Althanstr. 14, Vienna, 1090 Austria

**Keywords:** Rainforest, Phosphorus, P metabolomic niche, Diversity, Metabolomics

## Abstract

**Background:**

The availability of soil phosphorus (P) often limits the productivities of wet tropical lowland forests. Little is known, however, about the metabolomic profile of different chemical P compounds with potentially different uses and about the cycling of P and their variability across space under different tree species in highly diverse tropical rainforests.

**Results:**

We hypothesised that the different strategies of the competing tree species to retranslocate, mineralise, mobilise, and take up P from the soil would promote distinct soil ^31^P profiles. We tested this hypothesis by performing a metabolomic analysis of the soils in two rainforests in French Guiana using ^31^P nuclear magnetic resonance (NMR). We analysed ^31^P NMR chemical shifts in soil solutions of model P compounds, including inorganic phosphates, orthophosphate mono- and diesters, phosphonates, and organic polyphosphates. The identity of the tree species (growing above the soil samples) explained > 53% of the total variance of the ^31^P NMR metabolomic profiles of the soils, suggesting species-specific ecological niches and/or species-specific interactions with the soil microbiome and soil trophic web structure and functionality determining the use and production of P compounds. Differences at regional and topographic levels also explained some part of the the total variance of the ^31^P NMR profiles, although less than the influence of the tree species. Multivariate analyses of soil ^31^P NMR metabolomics data indicated higher soil concentrations of P biomolecules involved in the active use of P (nucleic acids and molecules involved with energy and anabolism) in soils with lower concentrations of total soil P and higher concentrations of P-storing biomolecules in soils with higher concentrations of total P.

**Conclusions:**

The results strongly suggest “niches” of soil P profiles associated with physical gradients, mostly topographic position, and with the specific distribution of species along this gradient, which is associated with species-specific strategies of soil P mineralisation, mobilisation, use, and uptake.

**Supplementary Information:**

The online version contains supplementary material available at 10.1186/s12870-024-04907-x.

## Background

Tropical forests are characterised by high biodiversity and biomass despite growing in strongly weathered soils. All tropical rainforests tend to have high productivity, rapid nutrient turnover, highly weathered soil, and low soil pH [1]. Tropical regions, such as those in Africa, Asia, and South America, have distinct geological histories that underlie the high biodiversity [2]. The distribution of plant species and soils are highly variable at local scales within tropical regions [3]. Several mechanisms have been proposed to explain the coexistence of many plant species in small areas in tropical forest i.e. the high biodiversity observed in tropical forests [4, 5]. Many of these mechanisms include heterogeneous disturbances and systems of regeneration [6]. A diverse topography [7], species-specific defenses against herbivores [8], soil traits heterogeneity [9–11], and differences in nutrient availability [10, 12–14] are the other most frequently discussed mechanisms.

The amount of P and its availability limits the productivity of many terrestrial and aquatic ecosystems [15]. Stocks of total soil P, the chemical forms of P, and P availability in the soil change as ecosystems age and develop, and these transformations can strongly influence ecosystem properties [16–18]. In particular, biological productivity in wet tropical forests is frequently limited by the availability of soil P [19, 20]. Soil taxonomic studies (Soil Survey Staff 2006) classify most tropical forest soils as Oxisols, Ultisols, Alfisols, Inceptisols, or Entisols [21]. P in these soils usually occurs as organic P and as occluded inorganic forms as part of pedogenic minerals. Occluded forms of P are especially common in older soils (Oxisols, Ultisols, and Alfisols), which frequently have very low or negligible amounts of primary minerals in their profiles and low concentrations of orthophosphate, which is the chemical form of P directly available to plants [17, 22]. In contrast, organic P can account for a considerable proportion of total P in tropical mineral soils, accounting for an average of 29 ± 3% of the total P in equatorial forests and in Panama (soil organic P in lowland tropical forests) [23, 24].

The turnover of organic P is the primary source of P for microbes and plants (soluble inorganic phosphate ions) in tropical soils [25, 26], even though several plant-soil processes can make organic and inorganic/occluded P available to plants, such as mobilisation by roots [27] or the chemical reduction of iron-phosphate complexes under anaerobic conditions [28]. Organic P in forest soils is derived from fresh organic matter (leaf litter), microbial biomass, and non-microbial biomass. In general there is evidence suggesting rapid decomposition of leaf litter in the humid tropics (< 1 year; [29], despite other studies have observed slower rates of leaf litter decomposition (> 1 year) [30]. Litter nonetheless provides most of the P that supports growth in strongly weathered tropical soils. This phenomenon of “*direct nutrient cycling*” in tropical forests is characterised by the fast release of biologically available phosphate to roots and mycorrhizae by the decomposition of leaf litter and the consequent fast absorption of P by microbes and plants, which is so rapid that the plant-soil P cycle closes, nearly preventing P from being lost *via* leaching or sorption to iron and aluminium oxides [31].

Knowledge of the various chemical types of soil organic P and of how plants and microbes use these forms of P is essential to better understand the global cycling and use of P in the plant-soil systems of tropical rainforests. ^31^P nuclear magnetic resonance (NMR) is an excellent tool for studying soil organic P because it provides quantitative data and allows the comparison of the various chemical forms of P [32–35]. In fact, ^31^P NMR allows to discern different molecules where P is present, molecules with different metabolic function such as phosphate mono- and di-esters such as nucleic acids, ATP or acilglyrates involved directly in metabolism pathways, metabolism control and energy transfer from molecules such as orthophosphate, pyrophosphates and polyphosphates, which are involved in P storage. For example, ^31^P NMR in soil studies provides data on the proportion of diester P associated with changes in microbial P compounds [36, 37] and the ratio of monoester P to diester P, information needed to characterise the lability and rate of turnover of soil organic P.

In these conditions of high species diversity under strong P limitation, we hypothesised, as suggested in previous reports [38], a long-term adaptation of sympatric species to maximise the capacity of taking up P while avoiding competition. We tested this hypothesis using ^31^P NMR spectroscopy in a P metabolomics study to determine the allocation of P to different biological functions in the soil. Our objective was to investigate the variation in soil P profiles along topographic gradients in two tropical forests with highly diverse compositions of tree species for determining whether changes in the profiles of soil organic P were correlated with changes in the composition of tree species and/or with distinct topographic environments and sites. We hypothesised that tree species would use P in specific ways, because the species occupy different functional [39] and biogeochemical [40] niches. Biogeochemical niche hypothesis captures niche parameters through species-specific elemental composition and stoichiometry [40–43]. The assumptions underlying it are based on the idea that each species is a unique genetic pool of individuals, a product of long-term evolutionary processes, so each species should have a specific structure and functionality (from gene expression to physiological processes). Since the fundamental biological processes (e.g. growth, secondary metabolism, reproduction and storage) have distinct rates in different species, depending on what selection has shaped, the different species have to allocate elements to various traits of tissues and organs differentially. The different use of bio-elements has proved to be even more different in sympatric species as a trait that could avoid direct competition [40]. Thus, as phosphorus is frequently limiting in tropical rainforests, we should expect that each species tends to have its own P-use strategy (P-metabolome niche) and that this should be underlying the higher species diversity of this high diverse ecosystem. We hypothesized that the tree species would use P in specific ways because each species occupies different functional [39] and biogeochemical [40] niches, thus providing species-specific litter that would accordingly modify the ^31^P-NMR profile of the underlying soil. Our specific expectations were: (1) species composition would influence the proportions of different P compounds in the underlying soil, and (2) the ^31^P NMR profiles would differ amongst and within sites (due to factors such as topography), indicative of differences in P use.

## Results

### 1D ^31^P NMR

**Fig. 1:**
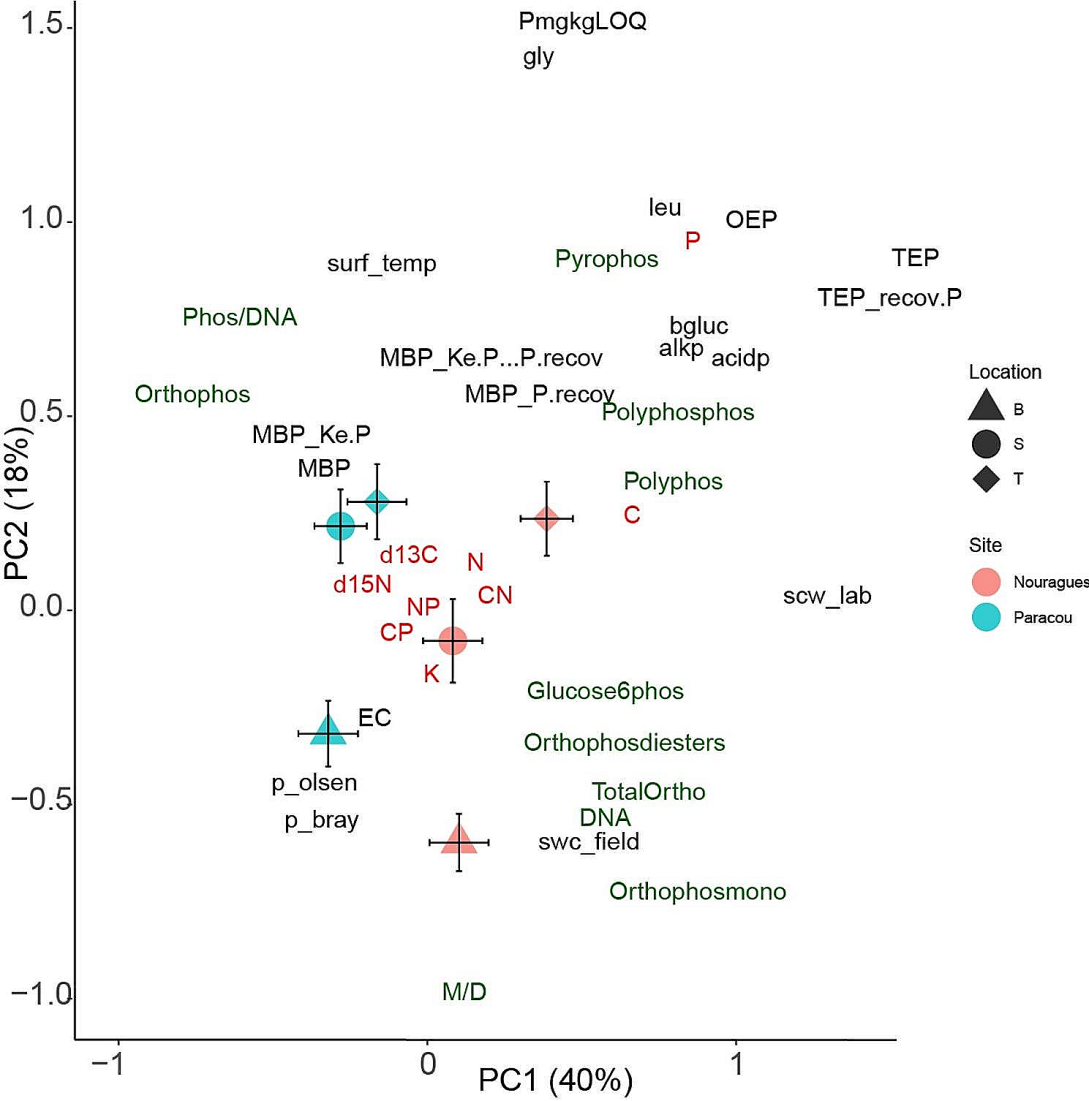
^31^P NMR chromatogram with the different molecules shown in the chromatogram

The spectra of acid-insoluble compounds indicated three main P resonances: monoesters, phospholipids (orthophosphate diester), and DNA. With NMR we were able to determine the family of P-compounds (Fig. 1), but we could not determine the exact compounds. Signals from nucleic acids (DNA: −0.37 ppm) and phospholipids were differentiated in the orthophosphate diester region and were readily identified in the soil samples. Inorganic and organic polyphosphates were differentiated by the presence of a signal at − 9 ppm from the α phosphate of organic polyphosphates. Some orthophosphate monoesters, such as mononucleotides derived from RNA and phosphatidyl choline, degraded rapidly to orthophosphate diesters in NaOH-EDTA, although DNA and phospholipids were more stable. The ^31^P NMR spectra of NaOH-EDTA extracts from all soil samples generally presented monoesters (∼ 25%) at 3.4 to 5.4 ppm, unhydrolysed diesters (∼ 20%) at − 1 to 2.3 ppm, and DNA (∼ 20%) at − 0.3 ppm. The less abundant forms were polyphosphate (∼ 10%) at − 5.6 to − 3.8 ppm, inorganic orthophosphate (hereafter ‘phosphate’; ∼10%) at 5.7 to 6.5 ppm, and the pyrophosphate inorganic form of P (∼ 5%) at 0.5 to 0.6 ppm. Glucose-6 phosphates had low resonance intensities, at 5.3 to 5.4 ppm.

### Soil P compounds and nutrients

**Fig. 2:**
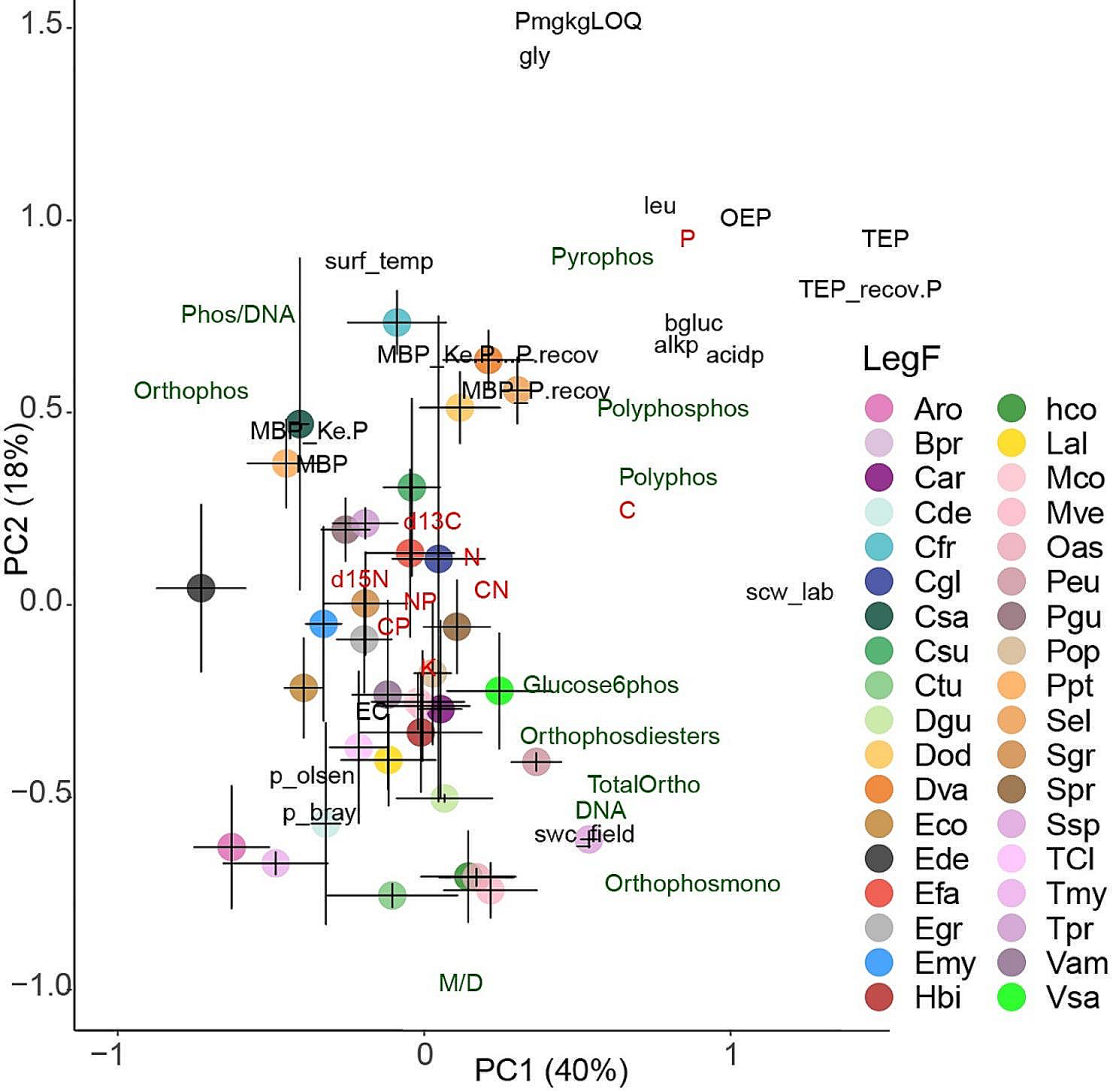
Principal component analyses of soil organic P compounds (green), nutrients (red), d^13^C, d^15^N, enzymatic activities, and ecophysiological variables (black) indicating significant effects of topographic position and site, with corresponding standard deviations Polyphos (polyphosphate); pyrophos (pyrophosphate) polyphosphos (polyphosphonate); orthophos (orthophosphate), orthophosmono (orthophosphate monosester), orthophosphate-diester (orthophosdiester); glucose6phos (glucose-6phosphates); SPAD—chlorophyll content; C—carbon; N—nitrogen; K—potassium; P—phosphorus. The abbreviations in the legend refer to the site (Paracou and Nouragues) and topographic position: T, top; S, slope; and B, bottom

In the PCA of organic P compounds; nutrients N, P, and K; enzymatic activity, and ecophysiological variables PC1 axis correlated best with site whereas PC2 correlates with samples of different topographic positions within each site (PC2) (Fig. 2). We examined the PCA results to identify the components that correlated best with site for better understanding the relationships of site with organic P compounds and the concentrations of C, N, P, and K. The PC2 axes correlated with topographic position and separated samples from the top, slope, and bottom positions, in Nourague and those of top and slope with respect to those of bottom in Paracou site.

The PCA results also indicated that soil with higher total P concentrations had higher concentrations of organic P compounds involved in storage (polyphosphates and polyphophos) and of C-free forms of P (orthophosphates and pyrophosphates). In contrast, soils with the lowest total P concentrations had higher concentrations of P compounds involved with genetic information, energy transfer, and protein anabolism such as orthophosphate monosester and orthophosphate-diester. Soils at the upper topographic positions had more total P (Table 1), which strongly correlated with the activities of acid and alkaline phosphatase, suggesting a greater “investment” of microbes and plants to allocation in P acquisition from soil when the level of total P is high. Tree characteristics also differed amongst the plots at the three topographic positions with wood density tending to be higher in the top plots whereas growth rate, and tree height tending to be higher in the bottom plots (Margalef et al. 2018). Each species occupied a different position in the 2D-plot of the PCA analysis as reinforced by the results of the PERMANOVAS (Table S3). The profiles of organic P compounds in the ^31^P NMR did not significantly vary between sites (Paracou vs. Nouragues; pseudo-F = 1.93; R^2^ = 0.015, *P* = 0.08), nor in function of topographic positions (top, slope, and bottom; pseudo-F = 1.73; R^2^ = 0.027, *P* = 0.568)) (Table S2). The interaction between topographic position and site was neither significant (pseudo-F = 5.68; R^2^ = 0.089, *P* = 0.195) (Table S2). Species had a significant effect on ^31^P NMR profile (pseudo-F = 3.09; R^2^ = 0.53, *P* < 0.001) (Table S3).

**Table 1:** Nutrient concentrations and stoichiometries and percentages of P compounds in the soil extracts

										P distribution, total P in extract (%)							
Material	Site	Location	Total P (mg g^− 1^)	Total C (mg g^− 1^)	Total N (mg g^− 1^)	C:N	C:P	N:P	K (mg g^− 1^)	Polyphos	Orthophos	Glucose6phos	Orthophosmono	Orthophosdiesters	DNA	Pyrophos	Polyphosphos
Soil	Paracou	Bottom	74.62	1.51	0.11	12.34	194	14.52	0.06	2.20	1.98	11.07	39.11	21.40	9.35	1.86	13.02
		Slope	118.83	2.28	0.16	14.06	203	14.32	0.08	6.58	7.83	17.69	22.87	24.37	10.79	0.00	9.87
		Top	67.22	1.87	0.14	13.53	290	21.40	0.06	0.72	1.20	13.52	40.23	35.91	4.42	0.00	4.00
	Nouragues	Bottom	58.36	2.43	0.18	13.05	424	32.68	0.20	2.16	10.65	3.88	39.77	37.67	3.42	0.42	5.03
		Slope	84.01	2.62	0.19	13.60	315	23.14	0.12	0.00	4.80	0.00	47.51	33.73	9.86	0.91	3.18
		Top	299.19	3.97	0.29	13.73	139	10.13	0.04	3.20	10.72	17.62	23.80	23.52	6.57	7.55	7.02

### P profile

The concentrations of the most abundant fractions of P (residual P, total P extractable by NaOH-EDTA, organic P, and inorganic P) varied amongst the soil samples, without any clear trends (Fig. 2). The total P concentration was 58–299 mg kg^− 1^; 56–88% was extracted by NaOH-EDTA, and the nonextracted fraction represented ‘residual P’. For the P extracted with NaOH-EDTA, 59–74% was organic and 26–41% was inorganic. Thus, most P was present in organic compounds than in inorganic forms suggesting a high biological use of P. Moreover, among these organic forms the highest proportion of P was found in molecules involved in active functions, such as growth, cellular control, or energy transfer. This is the case of the di-ester forms present in nucleic acid chains or storing energy molecules such as ATP.

The ratio of monoester to diester P was near 1, and diester-2 P was more abundant than diester-1 P, except for soils at the upper topographic position (in which the ratios of monoesters to diesters and diester-1 [phospholipids] to diester-2 [DNA and acid-unstable compounds] in the NaOH-EDTA extracts were similar).

## Discussion

### Species-specific utilization of phosphorus

**Fig. 3:**
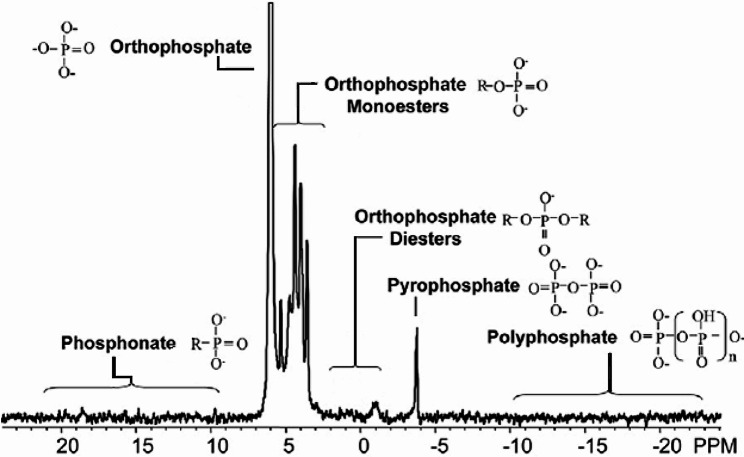
Principal component analyses of soil organic P compounds (green), nutrients (red), d^13^C, d^15^N, enzymatic activities, and ecophysiological variables (black) with species scores mean position, with corresponding confidence intervals (95%). Polyphos (polyphosphate); pyrophos (pyrophosphate) polyphosphos (polyphosphonate); orthophos (orthophosphate), orthophosmono (orthophosphate monosester), orthophosphate-diester (orthophosdiester); glucose6phos (glucose-6phosphates); SPAD—chlorophyll content; C—carbon; N—nitrogen; K—potassium; P—phosphorus. The abbreviations in the legend refer to the tree species: Aro, *Aniba rosaeodora*; Bpr, *Bocoa prouacensis*; Car, *Chrysophyllum argenteum*; Cde, *Capiro decorticans*; Cfr, *Catostemma fragrans*; Cgl, *Caryocar glabrum*; Csa, *Chrysophyllum sanguinolentum*; Csu, *Carapa surimensis*; Ctu, *Chimarrhis turbita*; Dgu, *Dicorynia guianensis*; Dod, *Dipteryx odorata*; Dva, *Drypetes variabilis*; Eco, *Eschweilera coriacea*; Ede, *Eschweilera decolorans*; Efa, *Eperua falcata*; Egr, *Eperua grandiflora*; *Emy, Eugenia*; Hbi, *Hirtella bicornis*; hco, *Hymanea courbaril*; Lal, *Licania alba*; Mco, *Moronobea coccinea*; Mve, *Micropholis venulosa*; Oas, *Oxandra asbeckii*; Peu, *Pouteria eugeniifolia*; Pgu, *Paloue guianensis*; Pop, *Protium opacum*; Ppt, *Pradosia ptychandra*; Sel, *Sloanea*; Sgr, *Sapotaceae grandiflora*; Spr, *Sterculia pruriens*; Ssp, *Sterculia speciose*; TCl, *Tovomita clusiaceae*; Tmy, *Tetragastris*; Tpr, *Talisia praealta*; Vam *Vouacapoua america*; and Vsa, *Vochysia sabatieri*

Our findings support the hypothesis that soil phosphorus (P) profiles differ among samples collected beneath different tree species. The soil samples associated with each tree species occupy distinct positions in a 2D plot representing the main functional groups of metabolic P molecules and nutrient concentrations in the soils (Fig. 3; Table 2, and table S4). Species accounted for over half of the total variance in 31P NMR data. These consistent results indicate a species-specific pattern of P uptake and utilization, as well as species-specific interactions between trees and microbes (including mycorrhiza) and P transformations specific to the microbes associated with each tree species. Additionally, we observed a clear gradient in P compounds from high anabolic energy to storage, with the highest concentrations of P compounds associated with anabolic energy in soils with the lowest total P concentrations.

**Table 2:** Nutrient concentrations and stoichiometries and percentages of P compounds in the soil extracts

								P distribution, total P in extract (%)							
LegF	Total P (mg g-1)	Total C (mg g-1)	Total N (mg g-1)	C:N	C:P	N:P	K (mg g-1)	Polyphos	Pyrophos	Orthophos	Glucose6phos	Orthophosmono	Orthophosdiesters	DNA	Polyphosphos
Aro	49.66	2.2	0.17	13.12	0.04	0	0.16	3.59	1.36	16.83	11.44	13.75	7.51	11.72	33.8
Bpr	116.24	3.36	0.24	14.03	0.02	0	0.05	16.42	11.34	13.11	12.67	14.59	5.58	8.73	17.57
Car	65.44	1.9	0.14	13.12	0.03	0	0.09	6.1	18.36	18.43	17.62	8.17	10.76	10.74	9.84
Cde	64.73	3.47	0.26	13.39	0.05	0	0.15	16.32	8.07	11.05	12.01	12.91	4.95	9.49	25.2
Cfr	412.47	3.44	0.24	14.07	0.01	0	0.05	8.68	10.86	18.66	13.61	12.65	5.57	7.31	22.67
Cgl	96.02	2.5	0.19	13.1	0.03	0	0.04	21	13.69	4.68	20.74	9.11	4.06	4.88	21.83
Csa	105.53	2.43	0.18	13.87	0.02	0	0.04	10.21	13.92	11.91	18.34	11.33	0.31	11.06	22.92
Csu	534.75	3.91	0.27	14.57	0	0	0.03	10.5	16.49	15.9	11.41	12.68	3.99	7.41	21.62
Ctu	65.96	2.38	0.18	13.36	0.04	0	0.12	14.53	8.16	16.44	8.32	15.95	5.18	11.35	20.1
Dgu	58.58	1.79	0.14	13.1	0.03	0	0.14	21	14.34	14.68	12.71	11.6	7.52	8.95	9.21
Dod	161.6	3.5	0.24	14.88	0.02	0	0.1	11.7	22.6	14.32	17.33	10.59	4.13	6.96	12.39
Dva	459.6	3.36	0.23	14.3	0.01	0	0.03	9.17	19.92	19.93	6.32	16.4	4.71	11.24	12.32
Eco	68.48	1.29	0.1	12.85	0.02	0	0.07	17.59	6.66	9.98	14.26	7	3.27	6.88	34.36
Ede	57.23	1.21	0.1	12.73	0.02	0	0.04	1.38	1.39	12.55	5.49	6.13	2.23	2.48	68.35
Efa	312.76	2.35	0.17	13.47	0.02	0	0.08	13.69	12.04	13.13	15.73	12.21	4.71	9.17	19.32
Egr	85.69	2.05	0.15	13.63	0.03	0	0.13	11.08	11.37	20.07	10.06	13.15	6.01	9.07	19.2
Emy	85.73	3.83	0.29	13.12	0.04	0	0.42	11.55	13.36	14.3	6.22	15.85	3.57	11.59	23.57
Hbi	53.99	1.88	0.15	12.91	0.03	0	0.14	4.87	10.21	6.55	22.21	12.73	2.07	11.87	29.49
hco	35.24	1.63	0.12	13.33	0.05	0	0.14	15.83	13.73	17.08	5.8	19.12	6.27	12.94	9.23
Lal	65.21	2.25	0.17	13.44	0.04	0	0.08	12.49	11.97	11.52	20.45	13.73	5.23	8.19	16.43
Mco	86.7	1.92	0.15	12.96	0.02	0	0.06	14.42	13.41	11.53	10.97	17.84	4.83	11.6	15.42
Mve	92.67	3.84	0.28	13.55	0.04	0	0.74	6.03	18.1	14.25	7.05	28.01	4.76	20.42	1.39
Oas	79.6	2.41	0.18	13.59	0.03	0	0.27	19.35	8.63	4.27	15.76	9.47	5.49	7.17	29.87
Peu	83.61	2.73	0.22	12.59	0.03	0	0.08	12.25	11.88	16.68	15.32	16.54	5.62	12.05	9.68
Pgu	75.54	2.46	0.18	13.61	0.03	0	0.03	12.46	12.67	21.37	13.8	8.68	5.13	6.47	19.43
Pop	58.48	2.05	0.15	13.29	0.04	0	0.08	15.99	11.4	22.33	13.14	7.8	4.69	6.78	17.89
Ppt	93.99	2.43	0.19	12.85	0.03	0	0.07	11.6	8.24	38.3	6.34	10.44	3.91	7.48	13.7
Sel	198.35	3.35	0.25	13.53	0.01	0	0.05	8.26	13.69	12.51	21.38	15.64	5.88	11.32	11.32
Sgr	170.26	2.02	0.15	13.13	0.02	0	0.07	12.45	16.32	13.96	9.52	12.2	4.89	7.26	23.39
Spr	100.52	3.26	0.24	12.83	0.01	0	0.04	14.46	13.69	9.88	16.36	14.06	4.78	9.66	17.14
Ssp	53.74	1.57	0.1	15.29	0.03	0	0.04	20.18	13.26	4.66	24.38	14.88	4.77	10.19	7.71
TCl	301.78	2.29	0.17	10.26	0.01	0	0.03	12.56	6.26	14.96	13.67	15.83	4.57	10.13	22.02
Tmy	45.54	1.21	0.11	10.75	0.03	0	0.11	18.18	4.73	12.19	16.99	13.88	4.54	11.25	18.25
Tpr	92.82	2.86	0.22	12.92	0.03	0	0.08	21.22	14.7	10.88	8.37	10.95	4.44	7.19	22.24
Vam	53.11	1.84	0.13	13.74	0.04	0	0.11	11.33	11.04	16.98	16.68	13.37	3.21	8.36	19.04
Vsa	91.05	2.97	0.21	13.94	0.03	0	0.04	17.69	14.2	3.24	20.13	14.88	5.3	10.01	14.56

Polyphosphates and polyphosphonates are chains of orthophosphates that serve as stored phosphate compounds. Our results revealed higher concentrations of orthophosphate, pyrophosphates, polyphosphonates, and polyphosphates in soils with higher total P concentrations and lower P availability at our study sites. Proportionally, more P was allocated to active metabolic molecules (e.g., DNA) and energy transfer (poly P, pyro P) in soils with lower total P concentrations and higher P availability. Higher concentrations of P storage compounds in the soil can be associated with low P availability, ensuring a controlled source of P for microbes when accessing soil P is challenging [44]. Moreover, the investment of microbes in building phosphatases was significantly lower in conditions of low carbon (C) and high poly P concentrations. This suggests that high polyphosphate concentrations may be associated with C conservation, particularly in sites where the soil has a high capacity to retain P. In these cases, higher polyphosphate reserves can serve as a P conservation trait, preserving C and providing a biological mechanism for accessing P from P reserves in microbial cells when necessary. Similarly, under conditions of very low C availability, higher PolyP levels may act as a compound for storing energy to conserve C [42].

Another study in the same ecosystem reported clear species-specific signatures in general foliar metabolomics profiles, indicating functional niches for dominant tree species in the region [43, 44]. Our results align with our initial hypothesis that the quantities of different P compounds would differ among soil samples due to interspecific differences in P utilization strategies and cycling. This finding is consistent with previous observations [45, 46]. For example, in a Panama rainforest, Condit et al. (2013) found that dry-season intensity and soil phosphorus were the strongest predictors, affecting the distribution of over half of a set of 550 tree species.

Our findings are also in line with previous studies that have identified associations between sets of species and specific habitats in tropical rainforests. The distribution of tree species is influenced by spatial variability in soil properties, such as nutrient availability and topography [47–50]. An extension of classical ecological niche theory, known as the “biogeochemical niche hypothesis“ [45], suggests that each species tends to reach an optimal chemical composition linked to a specific function that allows it to survive in its niche [39, 51, 52]. Our study provides evidence that organic P measurements can be used to characterize soil niches. The measurement and identification of different organic P molecules can serve as a powerful tool for characterizing most P-limited rainforests [23, 53]. In these cases, “P-metabolome niches” arise due to soil niche differentiation along natural spatial gradients of biotic and abiotic factors that influence P availability, total concentrations, and chemical forms of P, which, in turn, are associated with P utilization and cycling. Tropical trees may exhibit finely subdivided niches [54], although direct evidence for measurable variables of overall functional differences among sympatric species and geological processes is currently limited. However, our results clearly indicate that soil beneath each tree species possesses a specific soil P-metabolome. The distinct strategies of individual tree species for P uptake, litter quality, retranslocation, allocation, soil exudates, and biotic interactions with soil microorganisms and other tree species contribute to a micro-scale soil space with a unique P-metabolome profile.

## Topography and regional factors

In addition to the differences in P-metabolome profiles in soils associated with different tree species, we also observed relationships between P-metabolome profiles and other factors such as site and topographic position (Fig. 2). These relationships suggest that microsite variations in P-metabolome profiles are influenced by biotic conditions and associated with distinct soil traits, including texture, that vary across different topographic situations. Topography creates diverse soil P conditions, with higher total P concentrations and lower P availability at higher elevations, and vice versa at lower elevations. This finding is consistent with more conservative resource-use traits observed at higher elevations, such as the higher wood density of trees, and the opposite pattern at lower elevations. Furthermore, the spread of species along the 2D space generated by the two principal component axes was also associated with these findings. Thus, each species has developed a specific niche for highly efficient P utilization along the gradient, resulting in a distinctive distribution profile of P compounds that maximizes their use efficiency. These results also suggest that a “niche” of P profiles is associated with a physical gradient, primarily determined by topographic positions, and reflects species-specific strategies for mineralizing, mobilizing, utilizing, and acquiring soil P.

The abundance of organic P compounds, such as diesters and phosphonates, varied according to site and topographic position. Specifically, DNA and polyphosphate concentrations increased from the bottom to the top. DNA, polyphosphate, and phosphonates are abundant in soils with high microbial activity [31]. Site and topographic position explained some of the variance in the P profiles, likely due to clay content, which can form organomineral complexes and better retain organic material, including organic P. Variations in P profiles may also be influenced by metal oxides, as they can strongly occlude P, including organic P molecules. The occasional flooding of the bottom plots may have influenced the redox states of metal oxides (specifically iron and manganese oxides; aluminum oxides are less affected), which, in turn, could have affected the adsorption/desorption dynamics of P fractions in the soil.

Furthermore, the higher presence of mono- and diesters observed in bottom soils with higher sand content aligns with previous findings. A greater presence of monoester phosphorous compounds has been linked to the decomposition of organic matter rich in P metabolites, such as phosphatidylcholine and phospholipids [31, 55]. Similarly, higher soil concentrations of diester phosphates have been positively associated with higher concentrations of sand-sized fractions compared to silt and clay fractions [56, 57].

Significant variations were observed in the ratios of monoesters to diesters and the ratios of different diesters (diester-1 and diester-2; Table 1) across different parts of the 31P NMR spectra. Gressel et al. (1996) suggested that the correlation between monoester P and alkyl C in the organic horizons of a tropical forest soil indicates that the mineralization of monoester P fractions is linked to the decomposition of plant structural components, and that a significant portion of the monoester P in NaOH extracts may originate from hydrolyzed phospholipids derived from plants. The alkaline hydrolysis of phosphatidylcholine to monoesters is well-documented [31, 55, 58]. Soils in semi-arid northern Tanzania [59], enriched with diesters in sand-sized fractions relative to silt and clay fractions, likely derive these diesters from plants. Labile organic P compounds (e.g., phosphonates and diesters) and phosphate diesters (e.g., DNA and other diesters) often exhibit inverse relationships. For instance, diester P may increase while phosphonates decrease when comparing soils from a native savanna to Oxisol soil of an improved pasture in Colombia [36, 53]. Similarly, in a Spodosol soil of a spruce-fir forest, diester P (0 ppm) increased while unidentified diester compounds (1.5 to 2.5 ppm) decreased with increasing decomposition levels [46].

The top plots had higher concentrations of total P but lower levels of available P due to adsorption onto fine-grained particles, particularly highly reactive P oxides. This occlusion mechanism of P requires greater investments in microbial and root activities to acquire P, which is consistent with the higher activities of acid and alkaline phosphatases observed. However, the higher presence of molecules associated with P storage (orthophosphate, pyrophosphates, and polyphosphate inorganic chains) in these top soils is consistent with a more conservative strategy [47–51], where more P is stored within cells to support biological functions that require P, independent of P uptake from the soil. Furthermore, in top soils, the limited availability of soil P was associated with tree species characterized by higher wood density, typically exhibiting more conservative traits. These findings provide further evidence of the links between soil P status, species distribution, and their life strategies in this tropical rainforest [52–54].

In conclusion, our study reveals that variations in P-metabolome profiles in soils are not only influenced by different tree species but also by other factors such as site and topographic position. Topography creates distinct soil P conditions, which, combined with the spread of species along the gradient, leads to species-specific strategies for P utilization and uptake. Additionally, the abundance of organic P compounds varies with site and topographic position, influenced by factors such as clay content and metal oxides. The presence of mono- and diesters in soils correlates with organic matter decomposition and sand content. The ratios of different P compounds further highlight the complex dynamics of P cycling in the soil. Our findings contribute to a better understanding of the interactions between vegetation, soil, and P dynamics in tropical rainforest ecosystems.

## Conclusions

The P metabolomic profiles of soil samples collected at different sites under trees in French Guianese rainforests differed greatly, with very significant differences amongst soils collected beneath different tree species. This result is consistent with the ecological niche theory and the biogeochemical niche hypothesis (a correspondence of species with specific environmental conditions) in this highly diverse tropical ecosystem. Using ^31^P-metabolomic profiles to analyse the functions of different plant species in a community thus allowed us to identify different soil ^31^P NMR profiles associated with each tree species.

Our multivariate analyses of the soil ^31^P NMR metabolomics data notably indicated a trend to find higher soil concentrations of P biomolecules associated with low P use and high P storage under higher total P concentrations at the top sites, coinciding with tree species with more conservative strategies associated with denser wood and with a soil texture and mineral composition providing high P immobilization capacity. The bottom sites tended, instead, to have higher soil concentrations of P biomolecules associated with biological activity under lower total P concentrations, but higher P-availability, coinciding with tree species with less dense wood and a soil coarser texture.

Our combined analysis of soil elemental composition and P metabolomics provides an improved understanding of environmentally linked shifts in soil P concentrations and availability with different allocations of P for growth and other functions, such as storage, defense, reproduction, or resistance that stress the key role of this element in tropical rainforest functioning. As more P is available, more P is invested in active metabolism and functional activity and less in storage.

## Methods

### Study area

French Guiana is on the northeastern coast of South America between 2°10′ and 5°45′N and 51°40′ and 54°30′W. 97% of the region is covered by lowland wet tropical forest [55]. A pronounced dry season, characterised by < 100 mm precipitation per month, extends from September to November and is associated with the displacement of the intertropical convergence zone. Mean daily temperature is 25.8 °C and varies by only 2 °C throughout the year; daily temperatures vary by 7 °C during the rainy season and by 10 °C in the dry season [56, 57].

Field work was conducted at two sites of mature lowland tropical rainforest: the Paracou Research Station (5°18′N, 52°53′W) and the Nouragues Research Station (4°05′N, 52°40′W). Mean annual rainfall is 2990 mm at Nouragues and 3160 mm at Paracou [58], although the dry season is more severe at Paracou [58]. Three topographical locations were selected at each site: top of hills (top), middle of slopes at an intermediate elevation (slope), and bottom of slopes at a low elevation, immediately above a creek [59].

### Study plots

We established 12 plots of 0.25 ha at each site stratified by three topographic positions to account for the heterogeneous soil texture: top of the hills, slope and bottom of the valleys, thus with 4 plots in each topographic position. In each plot, we delimited a central 20 × 20 m quadrat where we marked five evenly spaced sampling points around which we focused all our measurements. This design thus contained a total of 120 sampling points (2 sites × 3 topographic positions × 4 replicate plots per topographic position × 5 sampling points in each plot). In each sampling point we annotated the nearest tree in function of the linear distance to the trunk base. The bottom plots at both sites had higher sand contents and lower clay contents than the top and slope plots [57]. All trees (diameter at breast height ≥ 10 cm) within the 0.25-ha plots were mapped, tagged, and identified to species or genus using herbarium vouchers for determining the richness of the tree species for each plot.

### Sample collection

Thus, we collected five soil samples per plot to a depth of 15 cm (Topsoil) in June (wet season) 2015. Each sample was collected using an auger/corer and was a composite of three borings near (< 2 m apart) each other. All voucher specimens were deposited in the Herbarium of International Center for Tropical Botany in Miami, FL 33,199 USA. A total of 120 samples were collected around the trees. We aliquoted 5 g of each sample for ^31^P NMR analyses that were immediately placed into a paper bag and frozen in liquid nitrogen before transporting the samples to the laboratory. The rest of soil sample was transported to the lab and stored in plastic zip bags at 4ºC until all the other analyses (within 4 weeks). Soil storage at 4ºC has been shown to keep enzymatic activity of tropical soils better than frozen samples. Fresh soil was sieved to 2 mm; for each sample one part was used for enzymatic activity analyses and the other part was dried 24 h at 105ºC for gravimetric water content. The other soil variables were analyzed from the same soil samples as those used in the ^31^P NMR analyses to determine the effect of the tree species.

### Environmental biotic and abiotic data

We compiled data for 28 variables describing the pools of soil nutrients, activities of extracellular enzymes, and aboveground tree-community data for each site to characterise the potential micro-environmental and biotic drivers. Nutrient concentrations and ratios, d^13^C, d^15^N, enzymatic activities, and ecophysiological variables are abbreviated as: C, N, P, C:N, C:P, N:P, K, d^13^C, d^15^N, leu (enzymes leucine), gly (enzymes glycine aminopeptidases), alkp (enzymes alkaline phosphatases), acidp (enzymes acid phosphatases), bgluc (enzymes *β-*glucosidase), p_olsen (amount of soil phosphorus available by Olsen test), p_bray (amount of soil phosphorus available by Bray test), scw_lab (laboratory surface water content), swc_field (field surface water content), surf_temp (soil surface temperature), EC (soil electric conductivity, mS/cm), MBP (microbial biomass P, µg P / g soil DW), MBP_Ke-P (microbial biomass P, µg P / g soil DW with factor KeP = 0.40), MBP_P recov (microbial biomass P, µg P / g soil DW with P-recovery factor), MBP_Ke-P + P recov (microbial biomass P, ug P / g soil DW with P-recovery factor and KeP), TEP (Total extractable P, µg P / g soil DW), TEP_recov P (Total extractable P, µg P / g soil DW with recovery factor P applied ), OEP (Organic extractable P, µg P / g soil DW), and PmgkgLOQ (P concentration in soil, mg/kg Limit of Quantification). All the acronyms used throughout the text are described in Table S1.

### Nutrient pools

We collected soil cores from the topsoil of each sampling point using a soil auger (4 cm in diameter and 15 cm in length; Van Walt, Haslemere, UK) to analyse the nutrient status. The samples were sieved to 2 mm and then freeze-dried (Alpha 1–2 LDplus, Martin Chirst Freeze Dryers, Osterode, Germany). Subsamples were pulverised in a ball mill (MM400, Retsch, Haan, Germany) for the analysis of elemental composition. We weighed 0.15–0.2 g of soil using an MX5 microbalance (Mettler Toledo, Columbus, USA) for determining the concentrations of total carbon (C) and nitrogen (N) by combustion coupled to an isotopic ratio mass spectrometer at the Stable Isotopes Facility (UC Davis, USA). Concentrations of total P and potassium (K) were determined by diluting 0.25 g of soil with an acid mixture of HNO_3_ (60%) and H_2_O_2_ (30% w/v) and digested in a MARS Xpress microwave oven (CEM Corporation, Matthews, USA). The digested solutions were then diluted to final volumes of 50 mL with ultrapure water and 1% HNO_3_. Blank solutions (5 mL of HNO_3_ with 2 mL of H_2_O_2_ but no sample biomass) were regularly analysed. The content of each element was determined using inductively coupled plasma/optical emission spectrometry (ICP-OES Optima 4300DV, PerkinElmer, Wellesley, USA). We used the standard certified biomass NIST 1573a to assess the accuracy of the biomass digestion and analytical procedures.

### Determination of activities of extracellular enzymes

We determined the activities of the extracellular enzymes β-glucosidase, leucine and glycine aminopeptidases, and acid and alkaline phosphatases (βgluc, leu, gly, acidP, and alkP, respectively) in all the 120 topsoil samples. The activities of these enzymes can serve as proximal variables of microbial nutritional metabolism and depend mostly on the interaction of the relationship between supply and demand with environmental kinetics [60]. Fresh subsamples of 2-mm sieved soil were stored in ziplock plastic bags and stored at 4 °C until analysis. We quantified the maximum potential activities of each enzyme by colorimetric assays using *p*-nitrophenylphosphate and *p*-nitroaniline derivative chromogenic substances. These enzymes are involved in the mineralization of C, N and P (Sinsabaugh and Shah, 2012). *β*-glucosidase participates in the decomposition of plant tissues, catalyzing the hydrolysis of 1–4 glucosidic bonds of labile cellulose (cellobiose and cellodextrins) to yield glucose. Leucine and glycine amino-peptidases cleave N-terminal residues from proteins and peptides. Acid and alkaline phosphatases release orthophosphate from organic P compounds like labile nucleic acids, phospholipids and inositol phosphates by the hydrolyzation of oxygen-P bonds.

### Soil microbial biomass P and soil extractable P

Phosphorus in the microbial biomass (MBP) was measured using the chloroform fumigation extraction method according to Brookes et al. (1982) and Brookes et al. (1985). Two subsamples (10 g fresh weight) of sieved soil were taken for each topsoil sample. One subsample was fumigated for 24 h with chloroform and extracted with 0.5 M of NaHCO_3_ (10:1 v:w) after 30 min shaking. The other subsample was directly extracted following the same protocol. The extracts were then filtered with Whatman 42 equivalent paper. Total P content in the extracts was determined after digestion of 2.5 g of aliquot with HNO_3_ in a microwave oven (MARS Xpress, CEM Corporation, Matthews, USA). The digested solutions were then diluted to a final volume of 50 mL with ultrapure water and 1% HNO_3_. Blank solutions were regularly analysed in parallel. P concentration in the digested samples and blanks was determined using inductively coupled plasma/optical emission spectrometry (ICP-OES Optima 4300DV, Perkin-Elmer, Wellesley, USA). The microbial biomass P content was calculated from the difference between fumigated and non-fumigated samples and expressed per unit of soil dry mass. Given that inorganic P molecules can absorb onto soil surfaces (organic matter and minerals) it is necessary to account for this effect during the extraction of P from soil, this is done through the application of an empirically determined “P-recovery factor”. Thus, a known amount of inorganic P (12.5 µg of inorganic P added as KH_2_PO_4_) was added to some of the controls to calculate the P-recovery factor in our soils. Microbial biomass was then calculated using the fumigated and non-fumigated samples corrected with the P-recovery coefficient (MBP_P.recov). Microbial biomass P was also corrected to take into account the efficiency of the fumigation (MBP_Ke.P), using the conversion factor KeP = 0.40 (Jenkinson et al. 2004). The factor KeP is an empirical coefficient used to relate the quantity of material solubilized by chloroform to the size of the original biomass, in this case it indicates that with fumigation 40% of the biomass-P is extracted as inorganic P. We have used all the microbial biomass P variables with and without corrections (MBP, MBP_P.recov, MBP_Ke.P and MBP_Ke.P…P.recov) in the analyses to give an overview of the methodological issues related to the quantification of extractable P in soils and microbes and to provide additional information for those readers with a background in soil biogeochemistry.

The total P concentration measured in non-fumigated bicarbonate-extracts was referred as the total extractable P (TEP) fraction. One aliquot of the non-fumigated extracts was used to determine the inorganic extractable P (P_Olsen) by Olsen’s method (Watanabe and Olsen, 1965). The organic extractable P (OEP) in the non-fumigated extracts was calculated as the difference between TEP and P_Olsen. All bicarbonate-extractable P fractions were expressed as µg of P per gram dry soil. Soil extractable P was also determined with Bray-P (P_Bray) acid fluoride extraction (Bray and Kurtz 1945) in oven-dried soil subsamples.

### Sample processing for ^31^P NMR analysis

The soils were frozen in liquid nitrogen, lyophilised, and stored in paper bags at -80 °C. The samples were ground with a ball mill at 1500 rpm for 3 min, and the fine powder was stored at -80 °C until extraction of the metabolites.

### One-dimensional ^31^P NMR

Conventional one-dimensional (1D) ^31^P NMR (Box 1) was used to quantify the main organic and inorganic forms of extractable P using NaOH-EDTA, including DNA, total diesters and monoesters, phosphonates, pyrophosphate, and polyphosphate. All the identification of the target P classes was based on chemical shifts and previous reported data (Turner et al. 2003; Vestergren et al. 2012). P was extracted by shaking 1.5 g of dry ground soil from each composite sample for 4 h in 30 mL of a solution containing 250 mM NaOH and 50 mM Na_2_EDTA (Cade-Menun and Preston 1996). The extracts were centrifuged at 14 000 *g* for 30 min, and the supernatant (23 mL) was frozen at − 80 °C overnight and then lyophilised. Lyophilisation yielded 750 ± 50 mg of material, 80 mg of which was redissolved in 640 µL (1:8 w/v) of a solution containing 530 µL of D_2_O, 10 µL of 14.2 M NaOD, and 50 µL of 16 mM methylene diphosphonic acid trisodium salt (MDPA, Sigma-Aldrich product number M1886). The MDPA served as a P reference for quantifying individual compounds, and each 50-µL spike contained 50 µg of P. The redissolved solution was vortexed for 2 min and centrifuged at 10 000 *g* for 5 min, and 560 µL was then transferred to a 5-mm NMR tube for 1D ^31^P NMR.

NMR spectra for ^31^P were obtained using an Avance III 600 MHz spectrometer (Bruker, Ettlingen, Germany) operating at 161.76 MHz. NaOH-EDTA extracts were analysed using a 3.9-µs pulse (90°), a relaxation delay time of 2.0 s, an acquisition time of 0.9 s, and broadband proton decoupling. We recorded 15 000 scans per sample, and the experimental time was 12.5 h. Spectra were processed with a line broadening of 2 Hz, and chemical shifts of signals were determined in parts per million (ppm) relative to an external standard (85% orthophosphoric acid, H_3_PO_4_). The main chemical types of P compounds were identified based on previously reported chemical shifts [32, 61]. Peaks were first identified using an automatic procedure for fitting peaks; peaks that were clearly visible were manually selected using TopSpin 2.0 NMR software (Bruker, Germany). Signal areas were calculated by the deconvolution and integration of individual peaks. Concentrations of P compounds (mg P kg^–1^ soil) were calculated using the known P concentration of MDPA spiked in the sample, and concentrations per weight of air-dried soil were given. All NMR spectra were processed using TopSpin 2.0.

Signal intensity in the ^31^P spectra was assigned to the different types of P by first integrating across the following regions of broad chemical shifts: -21.5 to -18.5 ppm for nonterminal polyphosphate (poly P), -5.3 to -4.8 ppm for pyrophosphate (pyro P), -4.8 to -4.0 for terminal poly P, -1.5 to 2.5 for diester-P, and 2.5 to 7 ppm for orthophosphate (ortho-P and monoester-P). Deconvolution was then used to determine the intensity of up to 16 resonances in the ortho-P and monoester-P regions. Deconvolution analysis began by manually identifying chemical shifts of peaks and shoulders. Peak chemical shifts varied only slightly amongst the samples. The orthophosphate signal shifted the most (range: 5.56–5.74 ppm), which shifted amongst soil samples, sites, species, and topographic positions. This variation was most likely due to slight differences in pH amongst the samples, because the orthophosphate peak is highly sensitive to variation in pH [62]. Thus, ^31^P NMR allows the determination of the family of P-compounds (Fig. 3), but not of the exact compounds.

^31^P nuclear magnetic resonance (^31^P-NMR) is an analytical NMR technique that allows the detection of both the concentration and the chemical form of the most abundant phosphorus isotope, ^31^P, in the analytical sample. In the concrete case of ^31^P this analytical tool allows to separately detect the intensity of the signal (proportional to concentration in the analyzed sample) of each molecular structure where the ^31^P is located. In this way, we can detect the most abundant types of compounds containing ^31^P in biological samples. We explored different aqueous/organic solvent methods to extract as many groups of compounds as possible and the preliminary results showed that using an aqueous solution allowed extracting most of groups compounds. Standard 1D ^31^P NMR was used to quantify concentrations of the main organic and inorganic P classes, including DNA, total orthophosphate diesters and monoesters, phosphonates, pyrophosphate and polyphosphate. Phosphorus was extracted by shaking 1.5 g of dry and ground soil from each composite soil sample in 30 mL of a solution containing 250 mM NaOH and 50 mM Na2EDTA (ethylenediaminetetraacetate) for 4 h (Cade-Menun and Preston 1996). Identification of the target P classes was based on chemical shifts and previous reported data [32, 63].

### Statistical analyses

The relationships of the P compounds with species, site and topographic position were identified by a PERMANOVA [64] of the NMR data for each soil sample. Euclidean distance, species identity, site, and topographic position were the fixed factors, and plot was a random effect, with 2000 permutations. Differences amongst species, site, and topographic position and amongst the P compounds most responsible for these differences were determined by comparing the areas of the different metabolite peaks normalized relatively to internal standards. Principal component analyses (PCAs) were used for processing the “omic” data sets together with the other potential influencing soil parameters, to detect the part of the variance explained by species [65]. Pearson’s correlation was used to identify the relationships of the 1D NMR measurements and the relationships between the concentrations of nutrients and organic P compounds. Error estimates are standard errors of the mean unless otherwise stated.

All statistical procedures were performed using R v 3.5 (www.r-project.org) with the SEQKNN, VEGAN, FACTOEXTRA, FACTOMINER, DPLYR, RANDOMFOREST, and MIXOMICS packages.

### Electronic supplementary material

Below is the link to the electronic supplementary material.

Supplementary Material 1

## Data Availability

All data generated or analysed during this study are included in this published article [and its supplementary information files].
