# Supplementary material for: Different profiles of soil phosphorous compounds depending on tree species and availability of soil phosphorus in a tropical rainforest in French Guiana
Source: BMC Plant Biol. 2024 Apr 12;24:278. doi: 10.1186/s12870-024-04907-x (PMC11010349; doi:10.1186/s12870-024-04907-x)
Supplement: Supplementary file 1 — Supplementary Material 1 [file 12870_2024_4907_MOESM1_ESM.docx]

# **Different profiles of soil phosphorous compounds depending on tree species and availability of soil phosphorus in a tropical rainforest in French Guiana.**

Albert Gargallo-Garriga^1,2,3*^, Jordi Sardans^1,2^, Joan Llusià^2,3^, Guille Peguero^1,2,4^, Elodie A. Courtois^4,5^, Clément Stahl^6^, Otmar Urban^3^, Pau Nolis^7^, Miriam Pérez-Trujillo^7^, Teodor Parella^7^, Andreas Richter^8^, Ivan A. Janssens^4^, Josep Peñuelas^1,2^.

## Supporting Information

## Table S1. Definition of the acronyms used in the text

| Acronyms | Names |
| --- | --- |
| Polyphos | Polyphosphate |
| Pyrophos | Pyrophosphate |
| Orthophos | Orthophosphate |
| Glucose6phos | Glucose-6phosphates |
| Orthophosmono | Orthophosphate monosester |
| Orthophosdiesters | Orthophosdiester |
| DNA | Deoxyribonucleic acid |
| Polyphosphos | Polyphosphonate |
| TotalOrtho | Total Orthophosphates |
| C | Soil C concentration |
| N | Soil N concentration |
| P | Soil P concentration |
| CN | Soil C:N concentrations ratio |
| CP | Soil C:P concentrations ratio |
| NP | Soil N:P concentrations ratio |
| K | Soil K concentration |
| d13C | Soil d^13^C |
| d15N | Soil d^15^N |
| leu | Leucine aminopeptidases activity |
| gly | Glycine aminopeptidases activity |
| alkp | Alkaline phosphatases activity |
| acidp | Acid phosphatases activity |
| bgluc | *β-*glucosidase activity |
| p_olsen | Amount of soil phosphorus available by Olsen test |
| p_bray | Amount of soil phosphorus available by Bray test |
| scw_lab | Laboratory surface water content |
| swc_field | Field surface water content |
| surf_temp | Soil surface temperature |
| EC | Soil electric conductivity |
| MBP | Microbial biomass P, ug P / g soil DW |
| MBP_Ke.P | Microbial biomass P, ug P / g soil DW with factor KeP = 0.40 |
| MBP_P.recov | Microbial biomass P, ug P / g soil DW with P-recovery factor |
| MBP_Ke.P...P.recov | Microbial biomass P, ug P / g soil DW with P-recovery factor and KeP |
| TEP | Total extractable P, ug P / g soil DW |
| TEP_recov.P | Total extractable P, ug P / g soil DW with recovery factor P applied |
| OEP | Organic extractable P, ug P / g soil DW |
| PmgkgLOQ | P concentration in soil, mg/kg Limit of Quantification) |

Table S2. Permanova with site and topography as fixed factors and plot as random factor

|  | Df | Sums Of Squares | Mean  Squares | F | R^2^ | P |  |
| --- | --- | --- | --- | --- | --- | --- | --- |
| Site | 1 | 207665 | 207665 | 1.9335 | 0.01525 | 0.08 | . |
| Location | 2 | 372398 | 186199 | 1.7336 | 0.02735 | 0.568 |  |
| Site:Location | 2 | 1221149 | 610574 | 5.6848 | 0.08969 | 0.195 |  |
| Residuals | 110 | 11814502 | 107405 |  |  |  |  |
| Total | 115 | 13615713 |  |  |  |  |  |

Table S3. Permanova with species as fixed factor and plot as random factor

|  | Df | Sums Of Squares | Mean  Squares | F | R^2^ | P |  |
| --- | --- | --- | --- | --- | --- | --- | --- |
| Species | 31 | 7250675 | 233893 | 3.0867 | 0.53252 | 0.001 | *** |
| Residuals | 84 | 6365038 | 75774 | 0.46748 |  |  |  |
| Total | 115 | 13615713 |  |  |  |  |  |

Table S4. Nutrient concentrations and stoichiometries and percentages of P compounds in the soil extracts.

| Acron. | | Species | | | Polyphos | | | Pyrophos | | | Ortho-phos | | Glucose  6phos | | | Ortho-  phosmono | | | | Ortho-  phosdiester | | | | DNA | | | Polyphos | | | TotalOrtho | | | M.D. | | | Phos.DO | |
| --- | --- | --- | --- | --- | --- | --- | --- | --- | --- | --- | --- | --- | --- | --- | --- | --- | --- | --- | --- | --- | --- | --- | --- | --- | --- | --- | --- | --- | --- | --- | --- | --- | --- | --- | --- | --- | --- |
| Aro | | *Aniba rosaeodora* | | | 3.59±0 | | | 1.36± 0 | | | 16.83±0 | | 11.44±0 | | | 13.75±0 | | | | 7.51±0 | | | | 11.72±0 | | | 33.8±0 | | | 19.23±0 | | | 0.72±0 | | | 0.64±0 | |
| Bpr | | *Bocoa prouacensis* | | | 16.42±7.55 | | | 11.34± 4.97 | | | 13.11±5.99 | | 12.67±7.83 | | | 14.59±3.22 | | | | 5.58±1.15 | | | | 8.73±3.05 | | | 17.57±4.74 | | | 14.31±4.12 | | | 1.04±0.08 | | | 0.67±0.11 | |
| Car | | *Chrysophyllum argenteum* | | | 6.1±8.34 | | | 18.36± 8.68 | | | 18.43±12.55 | | 17.62±0.88 | | | 8.17±1.23 | | | | 10.76±12.62 | | | | 10.74±0.37 | | | 9.84±1.48 | | | 21.5±12.25 | | | 0.44±0.19 | | | 1.03±1.21 | |
| Cde | | *Capiro decorticans* | | | 16.32±0 | | | 8.07± 0 | | | 11.05±0 | | 12.01±0 | | | 12.91±0 | | | | 4.95±0 | | | | 9.49±0 | | | 25.2±0 | | | 14.45±0 | | | 0.89±0 | | | 0.52±0 | |
| Cfr | | *Catostemma fragrans* | | | 8.68±6.14 | | | 10.86± 4.2 | | | 18.66±13.23 | | 13.61±6.09 | | | 12.65±5.11 | | | | 5.57±2.96 | | | | 7.31±2.13 | | | 22.67±7.46 | | | 12.88±4.92 | | | 0.97±0.11 | | | 0.73±0.23 | |
| Cgl | | *Caryocar glabrum* | | | 21±0 | | | 13.69± 0 | | | 4.68±0 | | 20.74±0 | | | 9.11±0 | | | | 4.06±0 | | | | 4.88±0 | | | 21.83±0 | | | 8.94±0 | | | 1.02±0 | | | 0.83±0 | |
| Csa | | *Chrysophyllum sanguinolentum* | | | 10.21±0 | | | 13.92± 0 | | | 11.91±0 | | 18.34±0 | | | 11.33±0 | | | | 0.31±0 | | | | 11.06±0 | | | 22.92±0 | | | 11.37±0 | | | 1±0 | | | 0.03±0 | |
| Csu | | *Carapa suri0mensis* | | | 10.5±6.11 | | | 16.49± 4.45 | | | 15.9±4.84 | | 11.41±9.12 | | | 12.68±5.67 | | | | 3.99±3.16 | | | | 7.41±2.62 | | | 21.62±8.47 | | | 11.4±5.08 | | | 1.11±0.04 | | | 0.54±0.45 | |
| Ctu | | *Chimarrhis turbi0ta* | | | 14.5±10.9 | | | 8.16± 7.4 | | | 16.44±0.8 | | 8.32±4.56 | | | 15.95±0.26 | | | | 5.18±3.69 | | | | 11.35±3.71 | | | 20.1±21.78 | | | 16.53±0.01 | | | 0.97±0.02 | | | 0.54±0.5 | |
| Dgu | | *Dicorynia guianensis* | | | 21±0.89 | | | 14.34± 8.13 | | | 14.68±4.09 | | 12.71±5.44 | | | 11.6±1.65 | | | | 7.52±3.01 | | | | 8.95±3.42 | | | 9.21±5.8 | | | 16.47±6.43 | | | 0.74±0.18 | | | 0.84±0.01 | |
| Dod | | *Dipteryx odorata* | | | 11.7±8.49 | | | 22.6± 6.34 | | | 14.32±6.46 | | 17.33±4.39 | | | 10.59±3.47 | | | | 4.13±0.99 | | | | 6.96±2.43 | | | 12.39±13.72 | | | 11.08±1.58 | | | 0.94±0.23 | | | 0.67±0.32 | |
| Dva | | *Drypetes variabilis* | | | 9.17±3.68 | | | 19.92± 2.76 | | | 19.93±6.09 | | 6.32±2.36 | | | 16.4±9.31 | | | | 4.71±2.36 | | | | 11.24±6.86 | | | 12.32±12.64 | | | 15.96±7.52 | | | 0.96±0.21 | | | 0.53±0.33 | |
| Eco | | *Eschweilera coriacea* | | | 17.59±12.3 | | | 6.66± 5.38 | | | 9.98±4.21 | | 14.26±5.74 | | | 7±2.22 | | | | 3.27±1.2 | | | | 6.88±2.7 | | | 34.36±23.37 | | | 10.15±3.89 | | | 0.71±0.09 | | | 0.48±0.02 | |
| Ede | | *Eschweilera decolorans* | | | 1.38±0 | | | 1.39± 0 | | | 12.55±0 | | 5.49±0 | | | 6.13±0 | | | | 2.23±0 | | | | 2.48±0 | | | 68.35±0 | | | 4.71±0 | | | 1.3±0 | | | 0.9±0 | |
| Efa | | *Eperua falcata* | | | 13.69±8.2 | | | 12.04± 4.51 | | | 13.13±5.34 | | 15.73±6.75 | | | 12.21±4.59 | | | | 4.71±1.88 | | | | 9.17±4.11 | | | 19.32±12.25 | | | 13.88±4.08 | | | 0.87±0.21 | | | 0.62±0.31 | |
| Egr | | *Eperua grandiflora* | | | 11.08±6.87 | | | 11.37± 5.15 | | | 20.07±19.27 | | 10.06±6.82 | | | 13.15±4.49 | | | | 6.01±2.53 | | | | 9.07±2.49 | | | 19.2±13.31 | | | 15.07±3.88 | | | 0.87±0.15 | | | 0.7±0.35 | |
| Emy | | *Eugenia* | | | 11.55±13.36 | | | 14.3± 0.33 | | | 6.22±0.93 | | 15.85±2.4 | | | 3.57±0.18 | | | | 11.59±1.44 | | | | 23.57±3.58 | | | 15.16±1.61 | | | 1.05±0.05 | | | 0.31±0.03 | | | 3.83±2.46 | |
| Hbi | | *Hirtella bicornis* | | | 4.87±1.98 | | | 10.21± 5.7 | | | 6.55±8.61 | | 22.21±1.81 | | | 12.73±12.89 | | | | 2.07±1.51 | | | | 11.87±9.57 | | | 29.49±20.9 | | | 13.95±11.09 | | | 0.8±0.29 | | | 0.19±0.02 | |
| hco | | *hymanea courbaril* | | | 15.83±0 | | | 13.73± 0 | | | 17.08±0 | | 5.8±0 | | | 19.12±0 | | | | 6.27±0 | | | | 12.94±0 | | | 9.23±0 | | | 19.21±0 | | | 1±0 | | | 0.48±0 | |
| Lal | | *Licania alba* | | | 12.49±7.62 | | | 11.97± 10.01 | | | 11.52±5.1 | | 20.45±7.61 | | | 13.73±4.21 | | | | 5.23±1.89 | | | | 8.19±2.58 | | | 16.43±11.27 | | | 13.42±3.5 | | | 1.03±0.2 | | | 0.68±0.26 | |
| Mco | | *Moronobea coccinea* | | | 14.42±1.75 | | | 13.41± 2.39 | | | 11.53±2.92 | | 10.97±8.55 | | | 17.84±7.13 | | | | 4.83±2.26 | | | | 11.6±4.09 | | | 15.42±12.9 | | | 16.43±4.91 | | | 1.07±0.16 | | | 0.46±0.31 | |
| Mve | | *Micropholis venulosa* | | | 6.03±0 | | | 18.1± 0 | | | 14.25±0 | | 7.05±0 | | | 28.01±0 | | | | 4.76±0 | | | | 20.42±0 | | | 1.39±0 | | | 25.18±0 | | | 1.11±0 | | | 0.23±0 | |
| Oas | | *Oxandra asbeckii* | | | 19.35±8.93 | | | 8.63± 11.5 | | | 4.27±3.65 | | 15.76±16.55 | | | 9.47±4.4 | | | | 5.49±0.03 | | | | 7.17±0.54 | | | 29.87±29.45 | | | 12.67±0.52 | | | 0.76±0.37 | | | 0.77±0.06 | |
| Peu | | *Pouteria eugeniifolia* | | | 12.25±8.85 | | | 11.88± 1.5 | | | 16.68±6.43 | | 15.32±1.74 | | | 16.54±0.71 | | | | 5.62±3.08 | | | | 12.05±5.14 | | | 9.68±0.59 | | | 17.67±2.06 | | | 0.94±0.07 | | | 0.58±0.5 | |
| Pgu | | *Paloue guianensis* | | | 12.46±13.29 | | | 12.67± 3.35 | | | 21.37±3.61 | | 13.8±3.66 | | | 8.68±1.49 | | | | 5.13±4.64 | | | | 6.47±1.39 | | | 19.43±2.06 | | | 11.61±3.25 | | | 0.76±0.08 | | | 0.89±0.91 | |
| Pop | | *Protium opacum* | | | 15.99±16.89 | | | 11.4± 6.26 | | | 22.33±2.84 | | 13.14±15.98 | | | 7.8±4.12 | | | | 4.69±3.46 | | | | 6.78±0.86 | | | 17.89±16.65 | | | 11.46±4.33 | | | 0.66±0.11 | | | 0.67±0.43 | |
| Ppt | | *Pradosia ptychandra* | | | 11.6±13.36 | | | 8.24± 4.31 | | | 38.3±24.61 | | 6.34±3.86 | | | 10.44±6.53 | | | | 3.91±1.82 | | | | 7.48±2.32 | | | 13.7±0.13 | | | 11.4±4.14 | | | 0.87±0.25 | | | 0.51±0.08 | |
| Sel | | *Sloanea sp* | | | 8.26±13.69 | | | 12.51± 4.32 | | | 21.38±10.42 | | 15.64±1.2 | | | 5.88±1.22 | | | | 11.32±1.31 | | | | 11.32±7.47 | | | 17.21±0.46 | | | 0.91±0.08 | | | 0.53±0.17 | | | 3.35±0.69 | |
| Sgr | | *Pouteria grandiflora* | | | 12.45±16.32 | | | 13.96± 9.83 | | | 9.52±5.92 | | 12.2±4.08 | | | 4.89±1.72 | | | | 7.26±2.42 | | | | 23.39±10.68 | | | 12.15±3.54 | | | 1±0.21 | | | 0.7±0.24 | | | 2.02±0.85 | |
| Spr | | *Sterculia pruriens* | | | 14.46±8.26 | | | 13.69± 4.43 | | | 9.88±7.7 | | 16.36±14.24 | | | 14.06±2.96 | | | | 4.78±2.34 | | | | 9.66±2.11 | | | 17.14±13.62 | | | 14.43±3.49 | | | 0.98±0.11 | | | 0.51±0.27 | |
| Ssp | | *Sterculia speciosa* | | | 20.18±2.88 | | | 13.26± 0.18 | | | 4.66±2.44 | | 24.38±0.73 | | | 14.88±0.22 | | | | 4.77±1.85 | | | | 10.19±1.62 | | | 7.71±0.35 | | | 14.96±0.24 | | | 0.99±0 | | | 0.49±0.25 | |
| TCl | | *Tovomita sp* | | | 12.56±6.26 | | | 4.6± 14.96 | | | 2.67±13.67 | | 2.07±15.83 | | | 3.09±4.57 | | | | 1.97±10.13 | | | | 1.44±22.02 | | | 7.4±14.7 | | | 1.29±1.09 | | | 0.27±0.47 | | | 0.25±2.29 | |
| Tmy | | *Tetragastris* | | | 18.18±4.73 | | | 12.19± 0 | | | 16.99±0 | | 13.88±0 | | | 4.54±0 | | | | 11.25±0 | | | | 18.25±0 | | | 15.79±0 | | | 0.88±0 | | | 0.4±0 | | | 1.21±0 | |
| Tpr | | *Talisia praealta* | | | 21.22±0 | | | 14.7± 0 | | | 10.88±0 | | 8.37±0 | | | 10.95±0 | | | | 4.44±0 | | | | 7.19±0 | | | 22.24±0 | | | 11.63±0 | | | 0.94±0 | | | 0.62±0 | |
| Vam | | *Vouacapoua america* | | | 11.33±4.7 | | | 11.04± 8.56 | | | 16.98±7.14 | | 16.68±6.11 | | | 13.37±6.29 | | | | 3.21±3.62 | | | | 8.36±2.67 | | | 19.04±16.28 | | | 11.57±4.96 | | | 1.13±0.14 | | | 0.37±0.39 | |
| Vsa | | *Vochysia sabatieri* | | | 17.69±12.69 | | | 14.2± 3.22 | | | 3.24±1.43 | | 20.13±17.86 | | | 14.88±11.79 | | | | 5.3±2.36 | | | | 10.01±3.12 | | | 14.56±17.78 | | | 15.31±5.48 | | | 0.89±0.45 | | | 0.52±0.07 | |
|  | | | | | | | | | | | | | | | | | | | | | | | | | | | | | | | | | | | | | |
| Acron. | | Species | | C | | N | | | P | | C:N | | C:P | | N:P | | | K | | | Delta^13^C | | Delta^15^N | | | Leu | | | Gly | | | AlkPhos | | | AcidPhos | | B-Gluc |
| Aro | | *Aniba rosaeodora* | | 2.2±0 | | 0.17±0 | | | 49.66±0 | | 13.12±0 | | 0.04±0 | | 0±0 | | | 0.05±0 | | | 26.68±0 | | 8.05±0 | | | 0.94±0 | | | 3.46±0 | | | 118.91±0 | | | 294.97±0 | | 5.16±0 |
| Bpr | | *Bocoa prouacensis* | | 3.36±1.68 | | 0.24±0.11 | | | 116.24±112.93 | | 14.03±0.99 | | 0.02±0.02 | | 0±0 | | | 0.09±0.01 | | | 28.24±0.66 | | 5.26±1.12 | | | 0.37±0.73 | | | 3.19±5.45 | | | 51.92±53.45 | | | 128.38±109.6 | | 2.74±5.48 |
| Car | | *Chrysophyllum argenteum* | | 1.9±0.81 | | 0.14±0.06 | | | 65.44±9.15 | | 13.12±0.31 | | 0.03±0.02 | | 0±0 | | | 0.15±0.04 | | | 28.42±0.09 | | 6.31±1.31 | | | 1.79±2.53 | | | 0±0 | | | 31.67±6.27 | | | 65.12±17.35 | | 0±0 |
| Cde | | *Capiro decorticans* | | 3.47±0 | | 0.26±0 | | | 64.73±0 | | 13.39±0 | | 0.05±0 | | 0±0 | | | 0.05±0 | | | 29.15±0 | | 4.46±0 | | | 0±0 | | | 0±0 | | | 16.27±0 | | | 53.03±0 | | 0±0 |
| Cfr | | *Catostemma fragrans* | | 3.44±1.22 | | 0.24±0.08 | | | 412.47±399.55 | | 14.07±0.46 | | 0.01±0.01 | | 0±0 | | | 0.04±0.02 | | | 28.46±0.08 | | 5.44±1.24 | | | 2.45±4.48 | | | 2.38±3.34 | | | 41.18±21.34 | | | 118.3±36.54 | | 1.5±2.41 |
| Cgl | | *Caryocar glabrum* | | 2.5±0 | | 0.19±0 | | | 96.02±0 | | 13.1±0 | | 0.03±0 | | 0±0 | | | 0.04±0 | | | 28±0 | | 4.42±0 | | | 0±0 | | | 0±0 | | | 33.12±0 | | | 74.23±0 | | 3.57±0 |
| Csa | | *Chrysophyllum sanguinolentum* | | 2.43±0 | | 0.18±0 | | | 105.53±0 | | 13.87±0 | | 0.02±0 | | 0±0 | | | 0.03±0 | | | 28.78±0 | | 4.74±0 | | | 0±0 | | | 0±0 | | | 18.11±0 | | | 66.83±0 | | 0±0 |
| Csu | | *Carapa surimensis* | | 3.91±1.42 | | 0.27±0.1 | | | 534.75±537.35 | | 14.57±0.31 | | 0±0 | | 0±0 | | | 0.12±0 | | | 28.85±0.63 | | 6.14±2.17 | | | 0.37±0.74 | | | 1.91±2.96 | | | 37.72±17.6 | | | 102.02±51.22 | | 2.6±5.21 |
| Ctu | | *Chimarrhis turbita* | | 2.38±0.63 | | 0.18±0.04 | | | 65.96±10.14 | | 13.36±0.85 | | 0.04±0 | | 0±0 | | | 0.14±0.04 | | | 28.05±0.45 | | 5.39±0.2 | | | 0±0 | | | 0±0 | | | 26.84±1.26 | | | 70.37±3.93 | | 0.47±0.66 |
| Dgu | | *Dicorynia guianensis* | | 1.79±0.59 | | 0.14±0.04 | | | 58.58±7.21 | | 13.1±0.94 | | 0.03±0.01 | | 0±0 | | | 0.1±0.14 | | | 28.43±0.16 | | 6.05±0.52 | | | 0±0 | | | 1.21±1.17 | | | 24.29±8.97 | | | 51.11±26.99 | | 1.58±2.23 |
| Dod | | *Dipteryx odorata* | | 3.5±1 | | 0.24±0.07 | | | 161.6±48.07 | | 14.88±1.71 | | 0.02±0.01 | | 0±0 | | | 0.03±0.08 | | | 28.25±0.34 | | 6.67±0.75 | | | 1.08±2.16 | | | 2.3±4.59 | | | 68.53±44.7 | | | 158.82±99.87 | | 0±0 |
| Dva | | *Drypetes variabilis* | | 3.36±1.32 | | 0.23±0.09 | | | 459.6±372.11 | | 14.3±0.74 | | 0.01±0.01 | | 0±0 | | | 0.07±0.01 | | | 21.55±14.37 | | 4.2±3.12 | | | 2.35±3.68 | | | 2.96±5.18 | | | 59.87±23.8 | | | 152.14±71.17 | | 4.45±3.95 |
| Eco | | *Eschweilera coriacea* | | 1.29±0.13 | | 0.1±0.01 | | | 68.48±29.52 | | 12.85±0.86 | | 0.02±0.01 | | 0±0 | | | 0.04±0.03 | | | 27.55±0.86 | | 6.3±0.9 | | | 0.18±0.31 | | | 1.24±1.76 | | | 34.06±11.53 | | | 98.94±33.66 | | 1.46±2.53 |
| Ede | | *Eschweilera decolorans* | | 1.21±0 | | 0.1±0 | | | 57.23±0 | | 12.73±0 | | 0.02±0 | | 0±0 | | | 0.08±0 | | | 28.4±0 | | 5.98±0 | | | 0±0 | | | 0.26±0 | | | 27.97±0 | | | 58.75±0 | | 0±0 |
| Efa | | *Eperua falcata* | | 2.35±0.95 | | 0.17±0.07 | | | 312.76±392.21 | | 13.47±1.03 | | 0.02±0.01 | | 0±0 | | | 0.13±0.04 | | | 28.23±0.21 | | 6.45±0.66 | | | 0.06±0.17 | | | 2.59±7.05 | | | 46.48±32.65 | | | 131.03±95.91 | | 2.64±3.45 |
| Egr | | *Eperua grandiflora* | | 2.05±0.41 | | 0.15±0.03 | | | 85.69±17.48 | | 13.63±1.2 | | 0.03±0.01 | | 0±0 | | | 28.18±0.08 | | | 28.22±0.27 | | 6.14±1.14 | | | 0.04±0.09 | | | 0.42±1.24 | | | 18.51±13.89 | | | 54.02±30.71 | | 0.28±0.63 |
| Emy | | *Eugenia* | | 0.29±0.17 | | 85.73±44.88 | | | 13.12±0.91 | | 0.04±0.01 | | 0±0 | | 0.42±0.27 | | | 0.14±0.09 | | | 5.95±0.03 | | 0±0 | | | 0±0 | | | 20.6±1.77 | | | 93.98±22.74 | | | 0.61±0.86 | | 2.04±0.34 |
| Hbi | | *Hirtella bicornis* | | 1.88±0.69 | | 0.15±0.06 | | | 53.99±7.08 | | 12.91±0.79 | | 0.03±0.01 | | 0±0 | | | 0.14±0.14 | | | 28.03±0.33 | | 6.62±0.35 | | | 2.06±2.92 | | | 0.9±1.28 | | | 20.62±13.72 | | | 44.65±18.44 | | 0.48±0.67 |
| hco | | *hymanea courbaril* | | 1.63±0 | | 0.12±0 | | | 35.24±0 | | 13.33±0 | | 0.05±0 | | 0±0 | | | 0.08±0 | | | 28.48±0 | | 3.7±0 | | | 0±0 | | | 0.28±0 | | | 25.86±0 | | | 67.95±0 | | 0±0 |
| Lal | | *Licania alba* | | 2.25±0.86 | | 0.17±0.05 | | | 65.21±24.8 | | 13.44±0.78 | | 0.04±0.02 | | 0±0 | | | 0.06±0.05 | | | 28.06±0.56 | | 5.96±1.11 | | | 0.47±1.42 | | | 0.76±1.05 | | | 31.51±14.81 | | | 74.19±31.87 | | 0.17±0.31 |
| Mco | | *Moronobea coccinea* | | 1.92±0.61 | | 0.15±0.05 | | | 86.7±13.35 | | 12.96±0.83 | | 0.02±0.01 | | 0±0 | | | 0.74±0.02 | | | 28.08±0.3 | | 6.28±1.25 | | | 0.4±0.8 | | | 0.21±0.39 | | | 23.42±19.38 | | | 85.23±74.14 | | 0.78±0.95 |
| Mve | | *Micropholis venulosa* | | 3.84±0 | | 0.28±0 | | | 92.67±0 | | 13.55±0 | | 0.04±0 | | 0±0 | | | 0.27±0 | | | 27.71±0 | | 7.27±0 | | | 0±0 | | | 0±0 | | | 40.9±0 | | | 76.45±0 | | 0±0 |
| Oas | | *Oxandra asbeckii* | | 2.41±0.31 | | 0.18±0.02 | | | 79.6±9.29 | | 13.59±0.42 | | 0.03±0.01 | | 0±0 | | | 0.08±0.3 | | | 28.17±0.27 | | 6.32±0.99 | | | 0.18±0.26 | | | 0.77±1.09 | | | 32.76±17.31 | | | 92.71±50.63 | | 0.88±1.24 |
| Peu | | *Pouteria eugeniifolia* | | 2.73±0.6 | | 0.22±0.04 | | | 83.61±7.75 | | 12.59±0.48 | | 0.03±0 | | 0±0 | | | 0.03±0.01 | | | 28.04±1.22 | | 6.95±1.58 | | | 1.88±2.66 | | | 4.98±4.8 | | | 60.11±11.64 | | | 155.58±29.94 | | 0±0 |
| Pgu | | *Paloue guianensis* | | 2.46±0.07 | | 0.18±0 | | | 75.54±15 | | 13.61±0.67 | | 0.03±0.01 | | 0±0 | | | 0.08±0 | | | 28.52±0.57 | | 7.01±0.19 | | | 0±0 | | | 0.71±1 | | | 27.98±9.59 | | | 76.97±23.27 | | 0±0 |
| Pop | | *Protium opacum* | | 2.05±0.43 | | 0.15±0.03 | | | 58.48±13.83 | | 13.29±0.16 | | 0.04±0.02 | | 0±0 | | | 0.07±0 | | | 28.07±0.02 | | 5.56±0.19 | | | 3.44±4.87 | | | 1.08±1.03 | | | 19.84±17.19 | | | 57.6±1.12 | | 0.39±0.55 |
| Ppt | | *Pradosia ptychandra* | | 2.43±0.06 | | 0.19±0 | | | 93.99±9.38 | | 12.85±0.09 | | 0.03±0 | | 0±0 | | | 28.43±0 | | | 27.71±1.05 | | 6.56±1.14 | | | 0±0 | | | 0±0 | | | 19.21±5.91 | | | 42.72±17.53 | | 0±0 |
| Sel | | *Sloanea sp* | | 0.25±0.05 | | 198.35±53.83 | | | 13.53±0.46 | | 0.01±0.01 | | 0±0 | | 0.05±0.02 | | | 28.03±0.49 | | | 6.54±0.61 | | 2.28±2.28 | | | 8.79±10.47 | | | 75.78±38.74 | | | 165.16±116.99 | | | 5.65±4.64 | | 2.59±0.48 |
| Sgr | | *Pouteria grandiflora* | | 0.15±0.06 | | 170.26±270.08 | | | 13.13±0.91 | | 0.02±0.01 | | 0±0 | | 0.07±0.07 | | | 0.04±0.68 | | | 5.65±1.48 | | 2.44±7.22 | | | 3.1±8.13 | | | 26.87±18.41 | | | 80.37±73.93 | | | 1.32±3.02 | | 2.43±0.92 |
| Spr | | *Sterculia pruriens* | | 3.26±2.2 | | 0.24±0.15 | | | 100.52±28.41 | | 12.83±1.89 | | 0.01±0 | | 0±0 | | | 0.04±0.02 | | | 28.47±0.44 | | 5.87±0.57 | | | 0.13±0.26 | | | 0.79±1.58 | | | 26.98±15.6 | | | 54.24±18.39 | | 0.03±0.05 |
| Ssp | | *Sterculia speciosa* | | 1.57±0.14 | | 0.1±0 | | | 53.74±0.46 | | 15.29±1.99 | | 0.03±0 | | 0±0 | | | 0.01±0.01 | | | 28.6±0.02 | | 6.43±0.85 | | | 0.09±0.12 | | | 1.43±1 | | | 22.15±9.84 | | | 60.74±21.88 | | 0±0 |
| TCl | | *Tovomita sp* | | 1.99±0.17 | | 0.14±301.78 | | | 465.92±10.26 | | 6.86±0.01 | | 0.02±0 | | 0±0.03 | | | 28.62±28.27 | | | 0.3±5.34 | | 0.82±0 | | | 0±0 | | | 0±33.46 | | | 25.11±93 | | | 74.4±1.56 | | 3.13±2.72 |
| Tmy | | *Tetragastris* | | 0.11±0 | | 45.54±0 | | | 10.75±0 | | 0.03±0 | | 0±0 | | 0.11±0 | | | 0.08±0 | | | 6.11±0 | | 0±0 | | | 0±0 | | | 25.69±0 | | | 76.64±0 | | | 0±0 | | 1.55±0 |
| Tpr | | *Talisia praealta* | | 2.86±0 | | 0.22±0 | | | 92.82±0 | | 12.92±0 | | 0.03±0 | | 0±0 | | | 0.11±0 | | | 28.1±0 | | 8.19±0 | | | 0±0 | | | 0±0 | | | 44.97±0 | | | 111.96±0 | | 0±0 |
| Vam | | *Vouacapoua america* | | 1.84±0.22 | | 0.13±0.02 | | | 53.11±18.62 | | 13.74±0.95 | | 0.04±0.01 | | 0±0 | | | 0.04±0.01 | | | 27.72±1.47 | | 7.14±1.16 | | | 0±0 | | | 0.09±0.17 | | | 41.25±19.17 | | | 90.72±55.96 | | 1.79±2.07 |
| Vsa | | *Vochysia sabatieri* | | 2.97±0.47 | | 0.21±0.02 | | | 91.05±7.27 | | 13.94±1.05 | | 0.03±0 | | 0±0 | | | ±0 | | | 28.03±0.58 | | 6.13±0.04 | | | 0±0 | | | 0±0 | | | 43.75±1.27 | | | 129.45±26.47 | | 0.5±0.71 |
|  | | | | | | | | | | | | | | | | | | | | | | | | | | | | | | | | | | | | | |
| Acron. | Species | | P-Olsen | | | | P-Bray | | | SWC.lab | | SWC.fied | | TEmp | | | EC | | MBP | | | MBP.Ke | | | MBP.P | | | MBPKeP…P | | | TEP | | | TEWPrec.P | | OEP | |
| PmgKg | *Aniba rosaeodora* | | 3.54±0 | | | | 2.1±0 | | | 61.03±0 | | 35.88±0 | | 24.48±0 | | | 1.13±0 | | 2.29±0 | | | 5.71±0 | | | 4.02±0 | | | 10.06±0 | | | 10.9±0 | | | 19.18±0 | | 4.85±0 | |
| Bpr | *Bocoa prouacensis* | | 2.35±0.44 | | | | 2.89±3.18 | | | 38.15±21.76 | | 31.59±6.3 | | 25.88±0.57 | | | 1.55±0.35 | | 1.01±0.69 | | | 2.53±1.73 | | | 1.66±1.02 | | | 4.14±2.55 | | | 8.01±2.8 | | | 17±11.58 | | 2.42±2.82 | |
| Car | *Chrysophyllum argenteum* | | 2.44±0.51 | | | | 1.33±0.09 | | | 23.18±0.19 | | 22.92±8.9 | | 23.65±0.87 | | | 1.34±0.21 | | 1.65±0.8 | | | 4.12±2 | | | 2.23±1.08 | | | 5.58±2.71 | | | 1.97±0.17 | | | 2.67±0.23 | | 0.4±0.24 | |
| Cde | *Capiro decorticans* | | 1.96±0 | | | | 1.55±0 | | | 28.44±0 | | 38.34±0 | | 23.87±0 | | | 1.06±0 | | 1.13±0 | | | 2.82±0 | | | 2.64±0 | | | 6.61±0 | | | 3.3±0 | | | 7.74±0 | | 0.56±0 | |
| Cfr | *Catostemma fragrans* | | 1.82±0.21 | | | | 1.29±0.59 | | | 41.43±7.9 | | 30.16±9.7 | | 25.63±2.12 | | | 1.38±0.25 | | 2.71±1.35 | | | 6.77±3.38 | | | 5.53±3.13 | | | 13.82±7.83 | | | 5.98±1.35 | | | 12.27±3.87 | | 2.15±0.74 | |
| Cgl | *Caryocar glabrum* | | 0.8±0 | | | | 0.44±0 | | | 38.52±0 | | 33.12±0 | | 23.4±0 | | | 1.21±0 | | 1.35±0 | | | 3.37±0 | | | 3.78±0 | | | 9.45±0 | | | 4.33±0 | | | 12.14±0 | | 1.7±0 | |
| Csa | *Chrysophyllum sanguinolentum* | | 2.92±0 | | | | 1.02±0 | | | 31.26±0 | | 34.68±0 | | 24.13±0 | | | 1.42±0 | | 1.23±0 | | | 3.07±0 | | | 2.88±0 | | | 7.2±0 | | | 2.94±0 | | | 6.88±0 | | 0.85±0 | |
| Csu | *Carapa surimensis* | | 2.05±0.6 | | | | 1.4±0.51 | | | 34.5±13.9 | | 33.21±2.4 | | 25.23±0.98 | | | 1.55±0.17 | | 1.94±0.56 | | | 4.84±1.41 | | | 4.06±1.21 | | | 10.15±3.02 | | | 7.48±3.75 | | | 15.96±8.66 | | 2.8±1.36 | |
| Ctu | *Chimarrhis turbita* | | 3.34±0.21 | | | | 2.6±0.24 | | | 29.85±0.35 | | 34.19±0.6 | | 24.02±0.02 | | | 1.4±0.23 | | 1.55±0.03 | | | 3.88±0.06 | | | 1.83±0.03 | | | 4.57±0.08 | | | 3.42±1.46 | | | 4.01±1.72 | | 0.2±0.29 | |
| Dgu | *Dicorynia guianensis* | | 1.89±0.48 | | | | 1.85±1.16 | | | 24.15±7.64 | | 38.63±7.8 | | 25.63±0.14 | | | 1.33±0.48 | | 1.22±1.16 | | | 3.04±2.9 | | | 1.63±1.16 | | | 4.08±2.91 | | | 4.77±2.09 | | | 8.33±6.19 | | 1.59±0.39 | |
| Dod | *Dipteryx odorata* | | 2.45±0.83 | | | | 1.12±0.8 | | | 46.86±13.33 | | 31.83±1.7 | | 26.04±1.82 | | | 1.44±0.31 | | 1.93±0.65 | | | 4.82±1.62 | | | 4.27±1.38 | | | 10.68±3.45 | | | 8.71±1.81 | | | 20.34±7.77 | | 3.93±1.1 | |
| Dva | *Drypetes variabilis* | | 2.13±0.98 | | | | 1.14±0.53 | | | 45.13±12.1 | | 28.86±5.5 | | 26.16±1.89 | | | 1.44±0.25 | | 1.97±1.03 | | | 4.94±2.57 | | | 4.11±2.18 | | | 10.26±5.44 | | | 8.72±5.07 | | | 20.14±14.99 | | 3.73±2.59 | |
| Eco | *Eschweilera coriacea* | | 1.94±0.75 | | | | 1±0.26 | | | 27.76±9.19 | | 30.72±4.2 | | 25.89±0.39 | | | 1.5±0.4 | | 2.33±0.58 | | | 5.82±1.46 | | | 3.52±1.07 | | | 8.81±2.68 | | | 3.86±0.7 | | | 6.07±2.72 | | 2.04±0.2 | |
| Ede | *Eschweilera decolorans* | | 1.97±0 | | | | 0.98±0 | | | 23.42±0 | | 34.39±0 | | 26±0 | | | 1.66±0 | | 2.69±0 | | | 6.71±0 | | | 3.44±0 | | | 8.59±0 | | | 3.32±0 | | | 4.24±0 | | 1.94±0 | |
| Efa | *Eperua falcata* | | 2.14±0.72 | | | | 1.2±0.47 | | | 39.43±15.66 | | 32.87±2.9 | | 25.88±1.55 | | | 1.44±0.28 | | 2.41±1.54 | | | 6.02±3.85 | | | 4.67±4.08 | | | 11.67±10.21 | | | 6.52±3.19 | | | 14.24±11.26 | | 2.8±1.63 | |
| Egr | *Eperua grandiflora* | | 2.75±0.55 | | | | 1.99±1.63 | | | 25.65±13.39 | | 29.13±5 | | 25.32±1.47 | | | 1.55±0.16 | | 2.41±0.74 | | | 6.03±1.86 | | | 4.54±1.96 | | | 11.35±4.9 | | | 4.95±2.33 | | | 10.51±7.39 | | 1.3±1.3 | |
| Emy | *Eugenia* | | 2.38±1.06 | | | | 32.86±4.47 | | | 36.2±1.06 | | 23.55±0.3 | | 1.01±0.34 | | | 2.3±0 | | 5.76±0.01 | | | 5.4±0.01 | | | 13.5±0.02 | | | 5.57±0.88 | | | 13.06±2.07 | | | 1.17±0.49 | | 56.14±3.75 | |
| Hbi | *Hirtella bicornis* | | 2.25±1.19 | | | | 1.23±0.17 | | | 24.95±8.73 | | 33.65±0.3 | | 23.97±0.66 | | | 1.47±0.05 | | 0.76±0.14 | | | 1.91±0.34 | | | 1.17±0.01 | | | 2.93±0.02 | | | 3.23±1.08 | | | 5.19±2.6 | | 1.13±0.1 | |
| hco | *hymanea courbaril* | | 3.33±0 | | | | 0.87±0 | | | 32.99±0 | | 35.97±0 | | 25.37±0 | | | 0.65±0 | | 0.65±0 | | | 1.63±0 | | | 1.33±0 | | | 3.31±0 | | | 3.84±0 | | | 7.82±0 | | 1.58±0 | |
| Lal | *Licania alba* | | 2.3±0.95 | | | | 1.43±0.7 | | | 31.98±3.55 | | 32.52±4.2 | | 25.08±1.42 | | | 1.33±0.28 | | 1.55±0.74 | | | 3.88±1.84 | | | 3.26±2.76 | | | 8.16±6.9 | | | 4.13±1.4 | | | 8.49±5.64 | | 1.17±0.69 | |
| Mco | *Moronobea coccinea* | | 1.67±0.69 | | | | 1.48±1.06 | | | 30.54±14.92 | | 33.25±2.2 | | 25.72±1.29 | | | 1.43±0.18 | | 2.28±1.52 | | | 5.71±3.79 | | | 4.82±3.73 | | | 12.06±9.33 | | | 4.44±2.27 | | | 9.82±7.8 | | 1.16±1.07 | |
| Mve | *Micropholis venulosa* | | 2.23±0 | | | | 1.93±0 | | | 35.94±0 | | 36.9±0 | | 23.97±0 | | | 1.32±0 | | 1.21±0 | | | 3.02±0 | | | 2.13±0 | | | 5.32±0 | | | 5.06±0 | | | 8.91±0 | | 1.3±0 | |
| Oas | *Oxandra asbeckii* | | 2.69±0.36 | | | | 1.1±0.61 | | | 31.5±10.88 | | 29.27±1.1 | | 25.43±1.27 | | | 1.69±0.23 | | 1.95±0.14 | | | 4.88±0.36 | | | 4.03±2.43 | | | 10.09±6.08 | | | 4.01±2.94 | | | 9.74±10.35 | | 1.21±1.6 | |
| Peu | *Pouteria eugeniifolia* | | 1.11±0 | | | | 0.98±0.01 | | | 42.73±4.26 | | 31.06±2.1 | | 24.3±0.71 | | | 1.53±0.12 | | 2.72±0.3 | | | 6.79±0.76 | | | 7.06±0.79 | | | 17.65±1.97 | | | 5.3±1.27 | | | 13.78±3.31 | | 1.63±0.62 | |
| Pgu | *Paloue guianensis* | | 1.62±0.03 | | | | 1.49±0.21 | | | 27.14±3.18 | | 27.59±1.3 | | 26.73±0.05 | | | 1.63±0.08 | | 0.84±0.25 | | | 2.1±0.63 | | | 1.02±0.31 | | | 2.55±0.76 | | | 2.94±0.05 | | | 3.58±0.06 | | 1.1±0.17 | |
| Pop | *Protium opacum* | | 1.18±0.1 | | | | 0.92±0.29 | | | 18.1±4.61 | | 24.69±5.1 | | 26.07±0.19 | | | 1.62±0.45 | | 2.06±0.17 | | | 5.14±0.42 | | | 2.63±0.21 | | | 6.58±0.53 | | | 2.59±0.07 | | | 3.32±0.09 | | 1.36±0.35 | |
| Ppt | *Pradosia ptychandra* | | 1.6±0.24 | | | | 1.24±0.46 | | | 17.83±9.85 | | 31.34±2.3 | | 25.5±0.14 | | | 1.6±0.06 | | 2.36±0.34 | | | 5.89±0.85 | | | 2.84±0.41 | | | 7.09±1.03 | | | 2.43±0.21 | | | 2.93±0.25 | | 0.9±0.28 | |
| Sel | *Sloanea sp* | | 1.06±0.38 | | | | 48.74±12.24 | | | 36.94±5.06 | | 26.71±1.27 | | 1.32±0.18 | | | 1.72±0.33 | | 4.29±0.82 | | | 3.94±0.52 | | | 9.84±1.3 | | | 9.61±2.92 | | | 23.84±12.09 | | | 4.43±1.41 | | 272.81±183.61 | |
| Sgr | *Pouteria grandiflora* | | 2.12±1.23 | | | | 32.52±14.24 | | | 31.86±3.52 | | 25.78±1.39 | | 1.48±0.21 | | | 3.07±1.57 | | 7.67±3.92 | | | 6.23±5.37 | | | 15.58±13.43 | | | 5.65±3.11 | | | 11.36±10.12 | | | 1.5±1.86 | | 123.84±86.49 | |
| Spr | *Sterculia pruriens* | | 2.13±1.73 | | | | 1.72±1.66 | | | 36.36±10.27 | | 34.47±3.4 | | 25.18±1.8 | | | 1.5±0.35 | | 1.2±1.38 | | | 2.99±3.46 | | | 1.97±1.59 | | | 4.92±3.98 | | | 4.54±1.1 | | | 10.43±4.2 | | 1.28±0.94 | |
| Ssp | *Sterculia speciosa* | | 1.87±0.11 | | | | 1.6±0.32 | | | 25.58±2.8 | | 29.29±0.3 | | 26.57±0 | | | 1.75±0.17 | | 0.71±0.67 | | | 1.77±1.69 | | | 0.86±0.82 | | | 2.15±2.05 | | | 2.98±0.76 | | | 3.62±0.93 | | 1.03±0.36 | |
| TCl | *Tovomita sp* | | 0.94±2.06 | | | | 1.47±37.69 | | | 17.23±32.9 | | 2.86±25.6 | | 1.35±1.64 | | | 0.1±5.75 | | 2.88±14.37 | | | 7.2±13.27 | | | 11.15±33.17 | | | 27.88±3.2 | | | 1.45±5.4 | | | 1.41±0.28 | | 0.21±193.4 | |
| Tmy | *Tetragastris* | | 0.75±0 | | | | 31.28±0 | | | 33.08±0 | | 24.87±0 | | 1.24±0 | | | 0.47±0 | | 1.18±0 | | | 0.96±0 | | | 2.4±0 | | | 3.97±0 | | | 8.08±0 | | | 1.78±0 | | 64.73±0 | |
| Tpr | *Talisia praealta* | | 1.94±0 | | | | 0.48±0 | | | 43.27±0 | | 30.57±0 | | 23.8±0 | | | 1.47±0 | | 2.71±0 | | | 6.77±0 | | | 7.59±0 | | | 18.99±0 | | | 5.29±0 | | | 14.83±0 | | 2.13±0 | |
| Vam | *Vouacapoua america* | | 2.19±0.81 | | | | 1.18±0.46 | | | 29.94±22.0 | | 33.19±7.2 | | 25.76±1.67 | | | 1.43±0.18 | | 1.71±0.57 | | | 4.26±1.44 | | | 2.84±0.84 | | | 7.09±2.1 | | | 5.5±1.45 | | | 9.29±2.8 | | 2.36±0.74 | |
| Vsa | *Vochysia sabatieri* | | 0.94±0.2 | | | | 0.54±0.08 | | | 39.03±0.8 | | 31.61±0.9 | | 23.8±1.13 | | | 1.64±0.45 | | 1.57±0.46 | | | 3.92±1.16 | | | 4.4±1.3 | | | 10.99±3.2 | | | 5.25±1.48 | | | 14.71±4.16 | | 2.05±0.65 | |
